# NAD^+^-boosting therapy alleviates nonalcoholic fatty liver disease via stimulating a novel exerkine Fndc5/irisin

**DOI:** 10.7150/thno.53652

**Published:** 2021-02-25

**Authors:** Dong-Jie Li, Si-Jia Sun, Jiang-Tao Fu, Shen-Xi Ouyang, Qin-Jie Zhao, Li Su, Qing-Xi Ji, Di-Ynag Sun, Jia-Hui Zhu, Guo-Yan Zhang, Jia-Wei Ma, Xiu-Ting Lan, Yi Zhao, Jie Tong, Guo-Qiang Li, Fu-Ming Shen, Pei Wang

**Affiliations:** 1 Department of Pharmacology, School of Pharmacy, Second Military Medical University/Naval Medical University, Shanghai, China.; 2 Department of Pharmacy, School of Medicine, Shanghai Tenth People's Hospital, Tongji University School of Medicine, Shanghai, China.; 3 Tongji University School of Medicine, Shanghai, China.; 4 Department of Organic Chemistry, School of Pharmacy, Second Military Medical University/Naval Medical University, Shanghai, China.; 5 Institute of Translational Medicine, Shanghai University, Shanghai, China.; 6 School of Life Science, East China Normal University, Shanghai, China.

**Keywords:** Fndc5, irisin, NAD^+^, SIRT2, nonalcoholic fatty liver disease, physical exercise

## Abstract

**Rationale:** Nicotinamide adenine dinucleotide^+^ (NAD^+^)-boosting therapy has emerged as a promising strategy to treat various health disorders, while the underlying molecular mechanisms are not fully understood. Here, we investigated the involvement of fibronectin type III domain containing 5 (Fndc5) or irisin, which is a novel exercise-linked hormone, in the development and progression of nonalcoholic fatty liver disease (NAFLD).

**Methods:** NAD^+^-boosting therapy was achieved by administrating of nicotinamide riboside (NR) in human and mice. The Fndc5/irisin levels in tissues and blood were measured in NR-treated mice or human volunteers. The therapeutic action of NR against NAFLD pathologies induced by high-fat diet (HFD) or methionine/choline-deficient diet (MCD) were compared between wild-type (WT) and *Fndc5^-/-^* mice. Recombinant Fndc5/irisin was infused to NALFD mice via osmotic minipump to test the therapeutic action of Fndc5/irisin. Various biomedical experiments were conducted *in vivo* and *in vitro* to know the molecular mechanisms underlying the stimulation of Fndc5/irisin by NR treatment.

**Results:** NR treatment elevated plasma level of Fndc5/irisin in mice and human volunteers. NR treatment also increased Fndc5 expression in skeletal muscle, adipose and liver tissues in mice. In HFD-induced NAFLD mice model, NR displayed remarkable therapeutic effects on body weight gain, hepatic steatosis, steatohepatitis, insulin resistance, mitochondrial dysfunction, apoptosis and fibrosis; however, these actions of NR were compromised in *Fndc5^-/-^* mice. Chronic infusion of recombinant Fndc5/irisin alleviated the NAFLD pathological phenotypes in MCD-induced NAFLD mice model. Mechanistically, NR reduced the lipid stress-triggered ubiquitination of Fndc5, which increased Fndc5 protein stability and thus enhanced Fndc5 protein level. Using shRNA-mediated knockdown screening, we found that NAD^+^-dependent deacetylase SIRT2, rather than other sirtuins, interacts with Fndc5 to decrease Fndc5 acetylation, which reduces Fndc5 ubiquitination and stabilize it. Treatment of AGK2, a selective inhibitor of SIRT2, blocked the therapeutic action of NR against NAFLD pathologies and NR-induced Fndc5 deubiquitination/deacetylation. At last, we identified that the lysine sites K127/131 and K185/187/189 of Fndc5 may contribute to the SIRT2-dependent deacetylation and deubiquitination of Fndc5.

**Conclusions:** The findings from this research for the first time demonstrate that NAD^+^-boosting therapy reverses NAFLD by regulating SIRT2-deppendent Fndc5 deacetylation and deubiquitination, which results in a stimulation of Fndc5/irisin, a novel exerkine. These results suggest that Fndc5/irisin may be a potential nexus between physical exercise and NAD^+^-boosting therapy in metabolic pathophysiology.

## Introduction

Nonalcoholic fatty liver disease (NAFLD) is a burgeoning health problem that encompasses a broad clinicopathological spectrum ranging from simple steatosis to nonalcoholic steatohepatitis (NASH), fibrosis and ultimately, cirrhosis 1. As lifestyles have become increasingly sedentary and dietary patterns have changed, the worldwide prevalence of NAFLD continues to increase 2. Many metabolic disorders, including hyperglycemia, abdominal obesity, diabetes mellitus, insulin resistance, and adipokine abnormalities, have close and reciprocal connections with NAFLD pathogenesis 1,2. Moreover, accumulating evidence has revealed that NAFLD might be an independent risk factor for cardiovascular diseases such as atherosclerosis and coronary heart disease 3. Although NAFLD has been intensively studied by a large number of experimental and clinical investigations in previous years, its pathophysiology and mechanism are not fully understood. The therapeutic options of NAFLD are still also limited.

Nicotinamide adenine dinucleotide (NAD^+^), a well-known intracellular electron carrier that functions as an essential cofactor in oxidative phosphorylation, is required for hundreds of enzymatic reactions. This molecule plays key roles in almost all major biological processes, while several lines of evidence in recent years have uncovered an entirely different role of cellular NAD^+^ as a critical modulator of intracellular signaling transduction 4. The abundance of NAD^+^ in cells is elegantly controlled by many factors, including its biosynthetic enzymes such as nicotinamide phosphoribosyltransferase (NAMPT) 5 and nicotinamide riboside kinase 6, or NAD^+^ consumers such as type III protein lysine deacetylases sirtuins family 7, poly(ADP-ribose) polymerases 8 and cADP-ribose synthase 9. By controlling the NAD^+^-responsive signaling factors, NAD^+^ regulates a large number of key cell biology procedures raging from cell survival/death, differentiation, aging/lifespan, energy hemostasis, etc. Moreover, due to the depressed intracellular NAD^+^ pool in pathophysiologies conditions from aging and various diseases, stabilizing the NAD^+^ level by administrating NAD^+^-boosting molecules such as nicotinamide riboside (NR), nicotinamide mononucleotide (NMN) and extracellular vesicle-contained NAMPT, represents a promising therapeutic strategy to stimulate hematopoiesis 10 and extend lifespan 11-13. Moreover, they can treat obesity 14, diabetes 15, brain ischemic injury 16, noise-induced hearing loss 17, kidney disease 18, vascular aging 19, dilated cardiomyopathy 20, etc.

As NR is a natural substance in milk, it has been proposed to be safe in human 21,22. NR is well-tolerated and elevates NAD^+^ in healthy adults 23. Moreover, NR augments the skeletal muscle NAD^+^ metabolome in aged adults and induces anti-inflammatory signatures 24. However, there are also reports that indicate NR have limited effects on insulin sensitivity and whole-body glucose metabolism in insulin-resistant obese men 25-27. In line with these, NAMPT-deficient cells seemed to be able to maintain substantial NAD^+^ levels 28.

In liver, NAD^+^-boosting therapy reversed high-fat high-sucrose induced fatty liver in mouse model 29. NR enhanced liver regeneration and reduced steatosis following partial hepatectomy 30. Our group previously reported that NR pronouncedly corrected fatty liver phenotypes in both NAD^+^-deficient mice and high-fat diet (HFD)-fed mice 31. Some mechanisms were proposed to explain these observations. NR prevents and reverts NAFLD by inducing a SIRT1- and SIRT3-dependent mitochondrial unfolded protein response, triggering an adaptive mitohormetic pathway to increase hepatic β-oxidation and mitochondrial complex content 29,32.

The exercise-modulated peptides and nucleic acids released from skeletal muscle and other organs, which are also known as 'exerkines', have now been recognized as key protectors against metabolism-related and neurodegenerative disorders 33,34. Irisin is a 12-kDa exerkine released from skeletal muscle, liver and adipose into the circulation proteolytically cleaved from fibronectin type III domain containing 5 (Fndc5), a 26-kDa transmembrane protein 35. Fndc5/irisin drives thermogenic capacity of brown adipose tissue to improve glucose homeostasis and metabolic status via endocrine, paracrine and autocrine mechanisms 35,36. Several new investigations have given evidence for the protective role of Fndc5/irisin in Alzheimer's disease 37, pulmonary ischemia injury 38, bone remodeling 39 and hepatic steatosis 40. In this study, we performed a general screening for a group of circulating 'exerkines' in NR-treated mice with NAFLD induced by high-fat diet (HFD), and found that a novel exerkine Fndc5/irisin was reduced after 3-month HFD treatment but rescued by NR supplement. Thus, we tested whether Fndc5/irisin participates in the safeguard action of NAD^+^-boosting molecule against NAFLD using Fndc5 knockout (*Fndc5^-/-^*) mice and further, investigated how NAD^+^ boosting molecule modulates Fndc5/irisin in NAFLD.

## Methods

### Animals

C57BL/6J mice were purchased from Sino-British SIPPR/BK Lab Animal Ltd. (Shanghai, China). The global Fndc5 knockout mice (C57BL/6J-Fndc5^tm1cyagen^,* Fndc5^-/-^*, Serial Number: KOCMP-00230-Fndc5) in C57BL/6 genetic background were generated by Cyagen Bioscience Inc (Guangzhou, China) using CRISPR/Cas9 technology. A mixture of plasmids encoding Cas9 and Fndc5 guide RNA targeting exon 2 of Fndc5 gene was microinjected into the fertilized C57BL/6 eggs, and the embryos were planted into pseudopregnant recipients. Founder lines of successful mutation of the Fndc5 gene cluster were identified through PCR genotyping of tail DNA and DNA sequencing (Primer: 5'-CACTTGTCTGCTCTCTGGTTCTGT-3'). The length of deleted sequence is 984 bp. Male Fndc5 heterozygote mice and female Fndc5 heterozygote mice were crossed to obtain Fndc5 homozygote mice. For mouse genotype identification, genomic DNA was prepared from the tail tips and the Fndc5 knockout was identified by PCR amplification (F1: 5'-CACTTGTCTGCTCTCTGGTTCTGT-3'; R1: 5'-ACGATATATTCTGTGTCCTCCTC-3'; R2: 5'-TAAGGGGAAGGGTCTATGTGAGC-3') and immunoblotting. See **Figure 2A-D** for more information. All mice were bred and housed in temperature-controlled cages under a 12/12-h light/dark cycle with free access to water and chow. Animal care and experimental procedures were approved by the Laboratory Animal Management Committee of Second Military Medical University and performed in accordance with the Guide for the Care and Use of Laboratory Animals published by the National Institutes of Health (NIH publication 86-23 revised 1985) and ARRIVE guidelines.

### NAFLD mice model and NR treatment

Two NALFD models were used in this study. The first model is achieved by using a high-fat diet (HFD; Catalogue: D12492, 60% fat, Research Diets, New Brunswick, NJ). Eight-week old male mice were fed the HFD diet for 16 weeks. Another mouse model of MAFLD was established by feeding the 8-week-old male mice a methionine/choline-deficient diet (MCD) diet (A02082002B; Research Diets, NJ) for 4 weeks as described previously 41,42. Mice were fed a normal chow diet and pure water served as controls. For NR administration, the animals were fed a diet of pellets made from HFD powder mixed with a commercial GMP-grade NR (NIAGEN®, ChromaDex Inc., Irvine, CA) in a dose of 400 mg/kg/day for 12 weeks (**Figure S1A**). For NAFLD model in *Fndc5^-/-^* mice, eight-week old male *Fndc5^-/-^* mice and their littermates wild-type (WT) mice were genotyped and then fed with chow, HFD or HFD + NR respectively for 16 weeks.

### Treatment with recombinant Fndc5/irisin in NAFLD mice

For testing the potential therapeutic action of Fndc5/irisin on NAFLD in mice, NAFLD was induced by MCD diets in mice as described above. Then, the mice were subcutaneously infused with recombinant Fndc5/irisin with 6×His tag at the C-terminus (#Abs04509, Absin Bioscience Inc, Shanghai, China) using an Alzet Osmotic minipump (Alzet DURECT Co., Cupertino, CA) for 7 days. The pumps were routinely inserted subcutaneously on the back of mice. The NAFLD mice infused with saline water were used as control. The dose of recombinant Fndc5/irisin was 5 nmol/kg body weight per day 43. The recombinant Fndc5/irisin was produced by mammalian expression system and the target gene encoding Asp32-Glu143 in Fndc5 gene. The purity is greater than 95% as determined by SDS-PAGE (endotoxin < 0.1 ng/μg determined by LAL test). At the endpoint of treatment, the mice were sacrificed and blood was collected and the liver pathologies were examined.

### Treatment with SIRT2 inhibitor AGK2 in NAFLD mice

To test whether inhibition of SIRT2 affects the NR-induced anti-NAFLD effects in mice, the C57BL/6J mice were divided into five groups: Chow (control), NR, NAFLD, NAFLD+NR and NAFLD+NR+AGK2. The mice in NAFLD group were fed with MCD diets for 4 weeks to induce NAFLD. The mice in NAFLD+NR group were fed with MCD diets for 4 weeks and injected with NR (i.p., 400 mg/kg/day dissolved in saline water) during the last two weeks. The mice in NAFLD+NR+AGK2 group were fed with NAFLD diets for 4 weeks and injected with NR plus AGK2 (MedChem Express, Catalogue: HY-100578, i.p.) simultaneously during the last two weeks. The dose of AGK2 (1 mg/kg/day) was chosen according to a previous study 44. At the endpoint of treatment, all the mice were sacrificed. Plasma was collected and the liver pathologies were examined.

### Human study

Six healthy volunteers participated in the present study. They were informed of the study purpose, experimental procedures, and possible risks of the study, and they provided written informed consent. All experimental procedures were approved by the Ethics Committee of Shanghai Tenth People's Hospital affiliated to Tongji University and complied with the 1975 Declaration of Helsinki. For physical exercise intervention, the volunteers were aksed to run 3000 meters per day (no speed requirement) for 2 weeks. The blood (100 µl) was obtained from fingertip before and after the two-week exercise. One month later, they were asked to ingest a commercial GMP-grade NR for human use (NIAGEN®; 500 mg, bid; ChromaDex, Inc.) as described previously 23. The blood (100 µl) was obtained from fingertip before and after the two-week administration again.

### Glucose tolerance test (GTT) and insulin tolerance test (ITT)

GTT and ITT were applied to determine the insulin resistance in mice as described previously 31. For GTT, mice were fasted overnight (~12 hours) and intraperitoneally injected with glucose (2 g/kg body weight). Blood samples were collected from the tail at 0, 30, 60, 90,120, 150 and 180 minutes and the blood glucose levels were determined. For ITT, mice were fasted for 6 h (from 10:00 am to 4:00 pm) and then injected with human insulin (Novo Nordisk, Princeton, NJ) at 2 units/kg body weight. Blood samples were taken at 0, 30, 60, 90,120, 150 and 180 minutes to determine glucose levels. The blood glucose concentration was plotted against time and the area under curve (AUC) was calculated.

### Enzyme-linked immunosorbent assay (ELISA)

Blood was obtained, collected in thylenediamine tetraacetic acid-anticoagulant plastic tubes, centrifuged, and plasma were either freshly used for ELISA or immediately frozen in liquid nitrogen until analysis. The plasma concentrations of fibroblast growth factor-1 (FGF1, Abcam, Cambridge, UK, Catalogue: DY4686-05), FGF21 (R&D Biosystems, Minneapolis, MN, Catalogue: MF2100), resistin (Boster Biological, Huhan, China, Catalogue: EK0582), adiponectin (R&D Biosystems, Catalogue: MRP300), leptin (R&D Biosystems, Minneapolis, MN, Catalogue: MOB00), chemerin (Boster Biological, Huhan, China, Catalogue: EK0582), vaspin (Aviscera Bioscience, Santa Clara, CA), hepcidin (Bioss Biotechnology Co., Beijing, China, Catalogue: bsk00526), interleukin-6 (IL-6, R&D Biosystems, Minneapolis, MN, Catalogue: D6050) and C-X-C motif chemokine 10 (CXCL10, PeproTech, Rocky Hill, NJ, Catalogue: 900-K) were detected using commercial ELISA kits according to the manufacturer's instructions. We applied two well-established kits (#EK-067-29, Phoenix Pharmaceutical, St. Louis, MO; #AG-45A-0046Y, Adipogen, Liestal, Switzerland) to measure the plasma irisin concentrations.

### Plasmids, cell culture and transfection

The Flag-tagged wild-type Fndc5 (WT-Fndc5), K127/131R mutant-Fndc5 (mutant 1, MT1-Fndc5), K177R mutant-Fndc5 (mutant 2, MT2-Fndc5) and K185/187/189R mutant-Fndc5 (mutant 3, MT3-Fndc5) were generated by Genescript (Nanjing, Jiangsu Province, China). They were subcloneed to a pcDNA3.1 plasmid. The AML12 normal hepatocyte cell line and HepG2 hepatocellular carcinoma cell line were obtained from American Type Culture Collection. The AML12 hepatocytes were cultured in 10% FBS, 10 µg/ml insulin, 5.5 µg/ml transferrin, 5 ng/ml selenium, 40 ng/ml dexamethasone and 1% antibiotic-antimycotic. The HepG2 cells were cultured in DMEM/F12 medium supplemented with 10% FBS, 1% antibiotic-antimycotic and 1% glutamax. The cells were maintained in an incubator under 37°C and 5% CO_2_. Cells were trypsinized and expanded every 48 to 72 h. Two day before transfection, about 1×10^5^ cells were seeded onto 6-well plates and allowed to grow to 70% confluence. Plasmids or siRNA targeting to SIRT1-7 (Santa Cruz Biotechnology, Santa Cruz, CA) were transfected into the cells using Lipofectamine LTX reagents (Invitrogen, Carlsbad, CA) according to the manufacturer's instructions.

### Histopathological examination

Oil Red O staining was used to specifically detect lipids in liver tissue. Hepatic tissue was cut into small pieces and fixed in 4% paraformaldehyde for 4 hours and embedded in OCT (Leica Camera AG, Wetzlar, Germany). Frozen sections (8 µm thickness) were made on a cryostat and the samples were fixed with 4% paraformaldehyde for additional 30 min. The slides were washed in distilled water and stained with Oil Red O for 15 min. Next, the slides were counterstained with hematoxylin for 10 s to identify the nuclei. Masson trichrome staining was performed to evaluate hepatic fibrosis. For Masson trichrome staining, paraffin sections were stained with Masson's trichrome (Sigma) according to standard procedures 45. The stained slides were examined under light microscopy at 200× magnification. Specimens were scored by red (Oil Red O) or blue (Masson's trichrome staining) using ImageJ software (Version 5.0, NIH).

### Immunohistochemistry and apoptosis assay

Immunohistochemistry was performed as described previously 46,47. The following antibodies were used: F4/80 (LS-C96373-100, Lifespan, 1:500 dilution), CD11b (ab6672, Abcam, 1: 1000 dilution) and α-SMA (ab7817, Abcam, 1: 500 dilution). Immunofluorescence TUNEL assay was used to assess apoptosis. Tissue sections were placed and fixed in 4% paraformaldehyde, and incubated with immunofluorescent TUNEL reaction mixture for 1 h in box. DAPI was used to stain nuclei. Immunofluorescence images were obtained in Olympus IX71 microscope (Tokyo, Japan, Leica).

### Immunoblotting

Immunoblotting was performed as described previously 48,49. Specific primary antibodies were listed in **Table S1**. The images were obtained with Odyssey Infrared Fluorescence Imaging System (Li-Cor). All immunoblotting experiments were repeated at least three times.

### Immunoprecipitation

Immunoprecipitation was performed as described previously 50. Cells were lysed in radioimmunoprecipitation assay buffer and the lysates were centrifuged at 13,000 rpm for 20 min. The supernatant was subjected to immunoprecipitation with protein A/G-agarose beads (Santa Cruz Biotechnology) and followed by immunoblotting analysis with specific antibodies or control IgG.

### Acetylation assay

To detect the acetylation signal, tissues or hepatocytes were lysed by RIPA buffer on ice. Tissue or cell extracts was immunoprecipitated using the monoclonal antibody against PGC-1α or Flag. After incubating with primary antibody against acetylated lysine and following secondary antibody, the acetylation of target proteins was detected. Meanwhile, the total protein amount of house-keeping gene such as GAPDH in the whole extract (input) were detected and used as loading controls.

### RNA isolation and Real-Time PCR

Total RNA was extracted from liver tissues in a single-step involving the use of TRIzol reagent (Invitrogen Life Technologies, Carlsbad, CA) according to the manufacturer's protocol. One microgram of RNA was reverse-transcribed to cDNA using the M-MLV enzyme (Promega, Madison, WI). Real-time quantitative PCR was performed using the ABI7500 real-time PCR detection system (ABI System) and SYBR® Green Real-Time PCR Master Mixes (ABI System) with specific primers. The primers were listed as in **Table S2**. The fold change in gene expression was determined using the 2^-∆∆^CT method. All samples were performed in duplicate. In the Real-Time PCR experiments, we judged the PCR quality according to the melting curve. If the melting curve was not acceptable, we excluded the data point.

### Determination of half-life of Fndc5

HepG2 cells were seeded in a 6-wells plate and serum-starved for 6 hours. Then, cells were exposed to NR (300 µM) under 150 μg/ml cycloheximide (Chx). Cells were harvested at different time points after Chx treatment, and cell extracts were immunoblotted with anti-Fndc5 and anti-Tubulin antibodies respectively. Bortezomib, lactacystin, MG132 and bafilomycin A1 were purchased from Selleck.

### Statistical Analysis

Data were analyzed with GraphPad Prism-8 statistic software (La Jolla, CA). All values are presented as the mean ± SEM. The values were analyzed by t-test (two groups) or One-Way ANOVA followed by Tukey *post-hoc* test (three groups or more). *P*<0.05 was considered statistically significant.

## Results

### NAD^+^-boosting molecules stimulates Fndc5/irisin in mice and human

**Figure 1:**
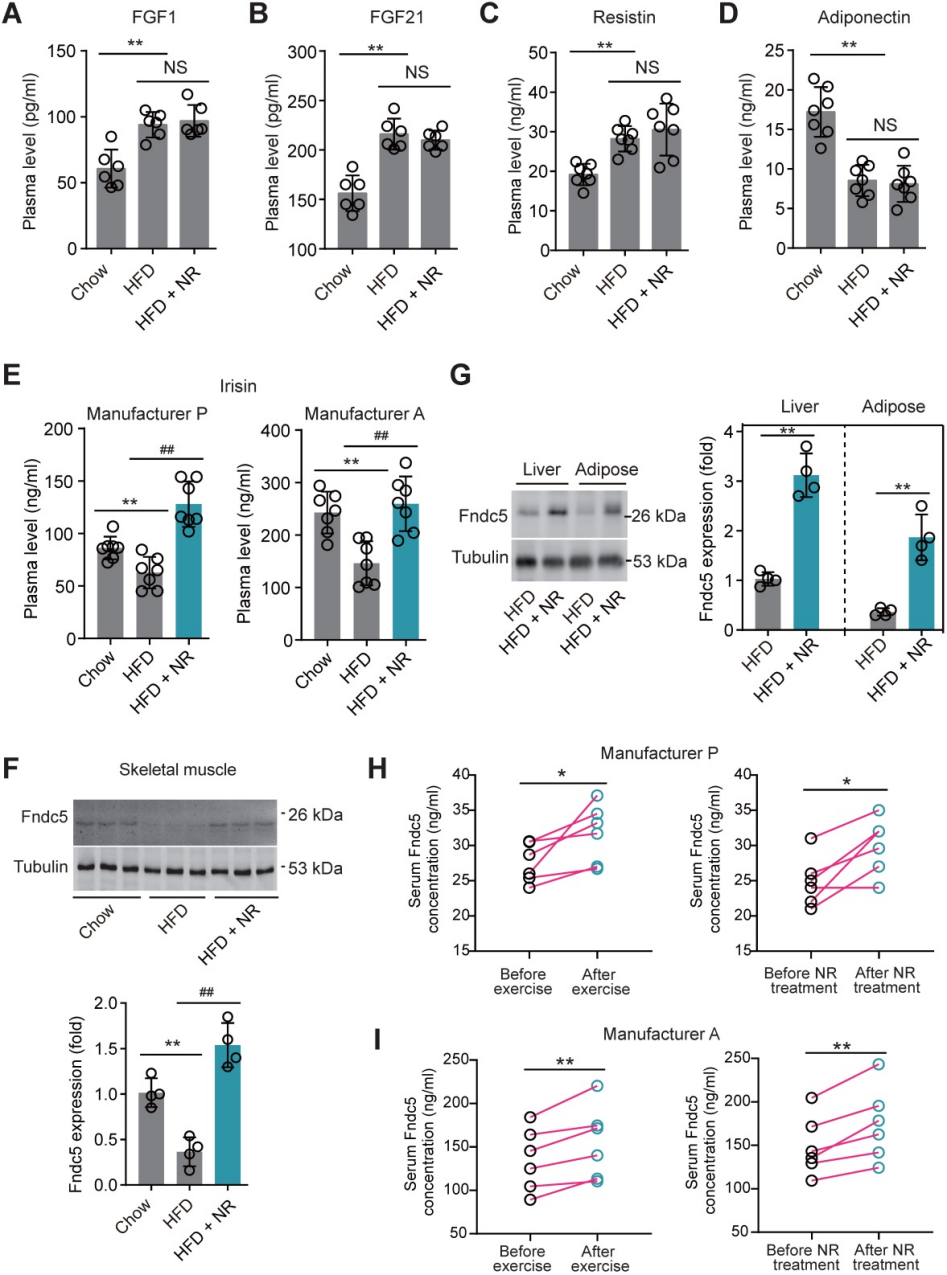
** Nicotinamide riboside, a well-established NAD+-boosting molecule, stimulates Fndc5/irisin in murine and human. (A-F)** The influence of NR treatment (400 mg/kg/d) on plasma concentrations of FGF1, FGF21, resistin, and adiponectin in HFD-induced NAFLD mouse model. FGF1, FGF21, resistin and adiponectin plasma concentrations were changed in NAFLD mice but not by NR. (**E**) Plasma irisin concentration was changed by NR treatment. **P<0.01 vs Chow, ^##^*P*<0.01 vs HFD, n = 8. NS, no significance. Two different commercial ELISA kits from Phoenix Pharmaceutical (Manufacturer P) and AdipoGen (Manufacturer A) were used. **(F)** The protein expression of Fndc5 in skeletal muscle of NAFLD mice with or without NR treatment. **P<0.01 vs Chow, ^##^*P*<0.01 vs HFD, n = 4. **(G)** The protein expression of Fndc5 in other tissues such as liver and adipose of NAFLD mice with or without NR treatment. **P<0.01 vs HFD by unpaired *t*-test, n = 4. **(H-I)** The influences of two-week NR supplement (500 mg, bid) or physical exercise on plasma irisin concentration in human volunteers detected by two different commercial ELISA kits from Phoenix Pharmaceutical (Manufacturer P, **H**) and AdipoGen (Manufacturer A, **I**). *P<0.05, **P<0.01 by paired *t*-test, n = 6.

To decipher the possible circulating exerkines mediating the NAD^+^-boosting therapy-induced beneficial metabolic modulation, we administrated NR in HFD-induced NAFLD model mice for 3 months (**Figure S1A**). At the end-point, we screened several circulating exerkines using ELISA assay in the three groups of mice (Chow, HFD and HFD + NR). We found the plasma concentrations of fibroblast growth factor-1 (FGF1), FGF21 and resistin were higher, whereas adiponectin concentration was lower, in HFD-fed mice than those in chow-fed mice (**Figure 1A-D**). However, NR treatment did not affect these alterations (**Figure 1A-D**). Plasma concentrations of leptin, chemerin, vaspin, hepcidin, IL-6 and CXCL10 were not different among the three groups in our setting (**Figure S1B-G**). Interestingly, we found that the plasma concentration of irisin was decreased in HFD group mice and significantly reversed by NR administration, which was confirmed by using two different commercial ELISA kits respectively (**Figure 1E**). This suggests that NR supplement affects irisin secretion. Although the absolute values of plasma irisin concentration differed between the two assays, the actions of NR were similar (**Figure 1E**). Correspondingly, irisin precursor Fndc5 protein expression in mouse skeletal muscle, which is the major source of released irisin, was decreased by HFD but upregulated by NR treatment (**Figure 1F**). As Fdnc5/irisin is not just a myokine but also secreted by other tissues such as adipose and liver 51,52, we additionally detected Fdnc5/irisin expression in adipose and liver. As expected, Fndc5 protein in mouse liver and adipose tissue was significantly elevated upon NR administration (**Figure 1G**).

We also compared the effects of physical exercise and NR supplement on blood irisin levels in six human volunteers using two different commercial ELISA kits from Phoenix Pharmaceutical and AdipoGen respectively. Both two ELISA kits showed that physical exercise for two weeks significantly elevated plasma irisin concentrations, while two-week NR supplement also enhanced plasma irisin concentrations to a similar extent in six health volunteers (**Figure 1H-I**). These results suggest that the NAD^+^-boosting molecule NR stimulates Fndc5/irisin in mice and human.

### Fndc5 is required for protection of NR against obesity and steatosis in NAFLD mice

**Figure 2:**
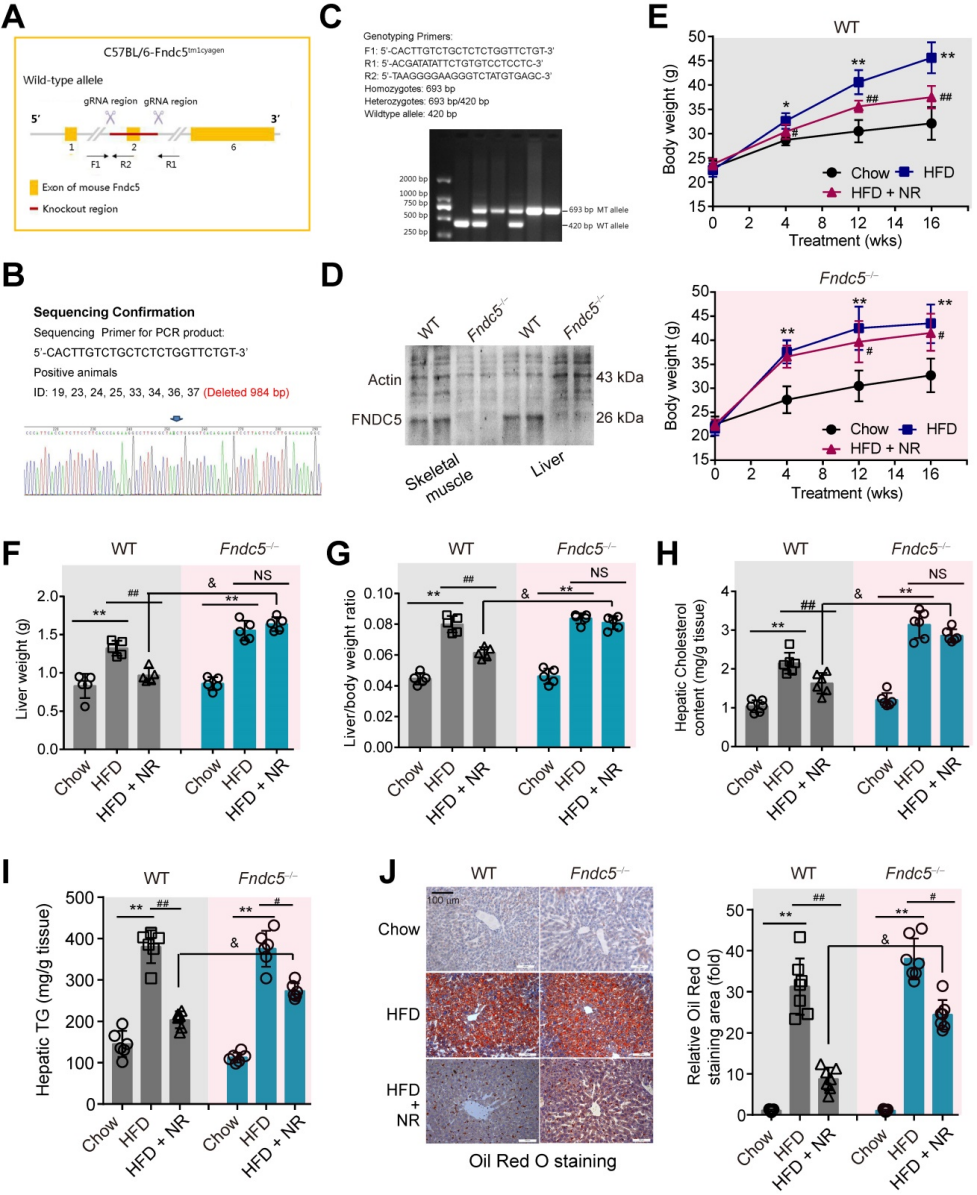
**Loss of Fndc5 attenuates the protection of NR against HFD-induced obesity and hepatic steatosis. (A)** Generation of global Fndc5 knockout mice by targeting exon 2 of Fndc5 using CRISPR/CAS9 technology-mediated deletion. **(B)** Deletion of Fndc5 exon 2 was confirmed using DNA sequencing. The sequencing results showed that 984 base pairs in the Fndc5 nucleotide sequence were deleted. **(C)** Genotyping and the used primers are showed. WT, wild-type; MT, mutant. (**D**) Deletion of FNDC5 protein in liver and skeletal muscle tissue. **(E)** The body curves of WT and *Fndc5^-/-^* mice fed with HFD and NR for 16 weeks**.** **P<0.01, *P<0.05 vs Chow, ^##^*P*<0.01, ^#^*P*<0.05 vs HFD, n = 6.** (F-G)** The liver weight **(F)** and the liver/body weight ratio **(G)** of WT and *Fndc5^-/-^* mice fed with HFD and NR for 16 weeks. **P<0.01 vs Chow, ^##^*P*<0.01 vs HFD, ^&^*P*<0.05* Fndc5^-/-^* vs WT, n = 6. NS, no significance. **(H-I)** Hepatic cholesterol (**H**) and triglyceride (**I**) levels in liver of WT and *Fndc5^-/-^* mice. *P<0.05 vs chow, ^##^*P*<0.01, ^#^*P*<0.05 vs HFD, ^&^*P*<0.05 *Fndc5^-/-^* vs WT, n = 6-7. NS, no significance. **(J)** Oil Red O staining showing the lipid accumulation (red staining) in liver of mice. **P<0.01 vs chow, ^##^*P*<0.01, ^#^*P*<0.05 vs HFD, ^&^*P*<0.05 *Fndc5^-/-^* vs WT, n = 6. NS, no significance. n = 6-7.

Considering that Fndc5 is widely expressed and can be secreted into blood to form irisin by various tissues 51,52, we generated a global knockout mice of Fndc5 (*Fndc5^-/-^* mice, **Figure 2A-C**) to test the role of Fndc5/irisin in protection of NR in NAFLD. In this mice strain, we confirmed the absence of Fndc5 protein in skeletal muscle and liver (**Figure 2D**). The wild-type (WT) and *Fndc5^-/-^* mice were fed with HFD for 16 weeks to induced NAFLD model. In WT mice, HFD-feeding resulted in a significant increase of body weight. In line with previous results from us 31 and other lab 14, NR remarkably attenuated the HFD-induced weight gain (**Figure 2E**). In *Fndc5^-/-^* mice, HFD induced more pronounced body weight gain, suggesting that Fndc5 knockout partly deteriorated the HFD-induced obesity. Strikingly, NR treatment reduced the body weight in HFD-fed *Fndc5^-/-^* mice to a much lesser extent compared with in WT mice. Similarly, NR administration decreased the HFD-induced increases of liver weight (**Figure 2F**) and liver weight/body weight ratio (**Figure 2G**) in WT mice but not in *Fndc5^-/-^* mice. The inhibitory actions of NR on serum lipid levels (**Table S3**) and liver cholesterol/triglyceride content (**Figure 2H**-**I**) were also significantly suppressed in *Fndc5^-/-^* mice. Accordingly, liver histopathology with Oil Red O staining showed NR treatment led to a sharper decrease of lipid content in WT mice compared with in *Fndc5^-/-^* mice (**Figure 2J**). We also determined the gene expression involved in lipid uptake (*low density lipoprotein receptor* [*LDL-R*] and *CD36*), transport (*ATP binding cassette subfamily G member 1* [*ABCG1*] and *ABCG5*), synthesis (sterol regulatory element binding transcription factor 2 [*SREBP-2]* and *acetyl-coenzyme A carboxylase* [*ACCα*]) and oxidation (*peroxisome proliferator activated receptor*-α [*PPARα]* and *acyl-CoA oxidase 1* [*Acox1*]). Loss of Fndc5 did not affect the effects of NR on *LDL-R, CD36, ABCG1, ABCG5, SREBP-2* and *ACCα*; however, it reduced the *PPARα* and *Acox1* mRNA expression change by NR (**Figure S2**), implying the action of NR on lipid oxidation may involve Fndc5. All these results suggest that deletion of Fndc5 compromises the protection of NR against HFD-induced obesity and hepatic steatosis.

### Deletion of Fndc5 diminishes improvement of NR on insulin resistance in NAFLD mice

**Figure 3:**
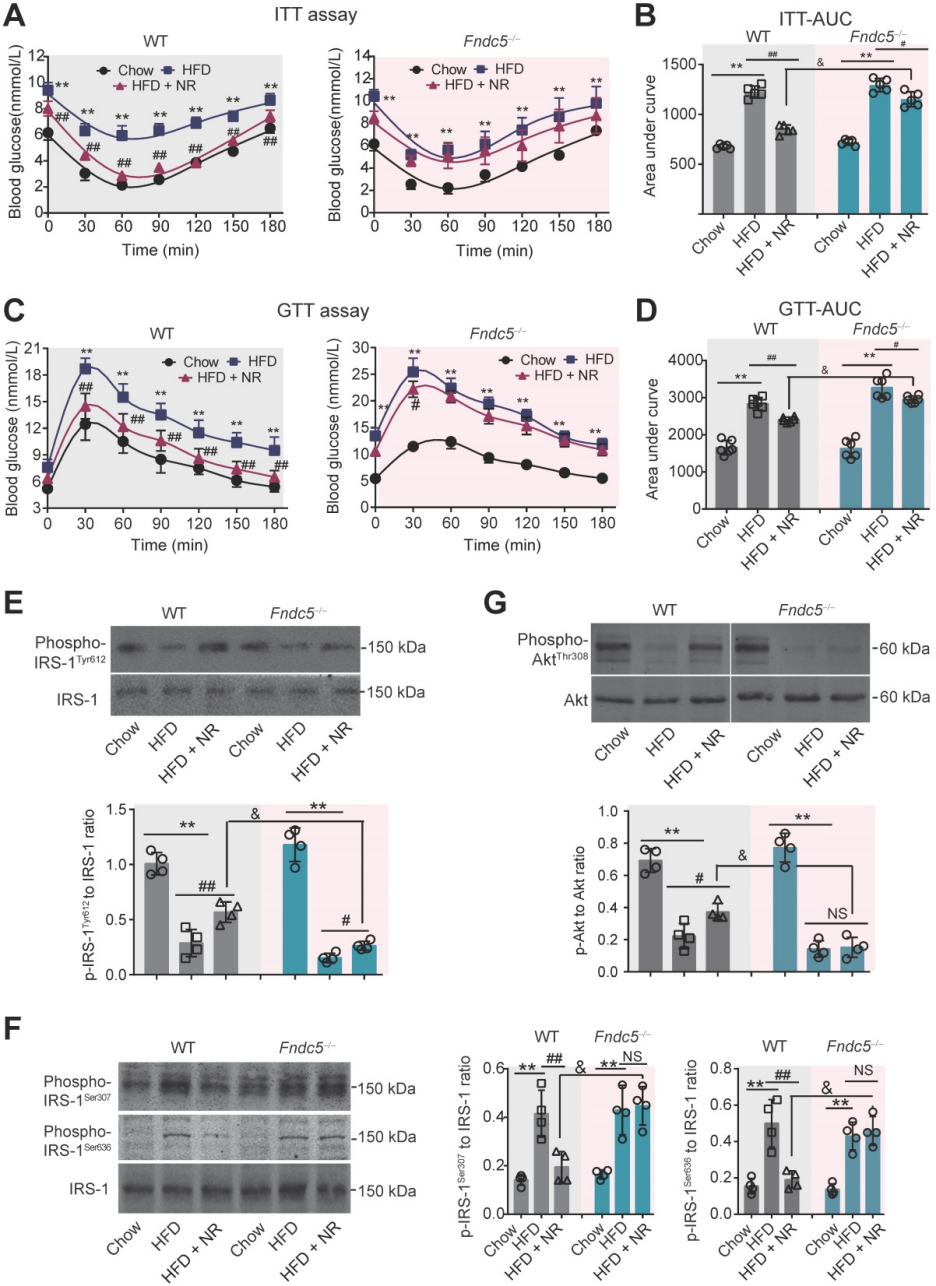
** Loss of Fndc5 diminishes the improvement of NR against HFD-induced insulin resistance. (A-B)** The ITT assay curve **(A)** and calculation of area under curve (AUC, **B**) on WT and *Fndc5^-/-^* mice. **(C-D)** The GTT assay curve **(C)** and calculation of AUC **(D)** on WT and *Fndc5^-/-^* mice. **(E-F)** The phosphorylation of IRS-1 at tyrosine site 612 of IRS-1 **(E)** and serine sites at 307 and 636 sites of IRS-1 **(F)** in liver tissue of mice were detected by immunoblotting. **(G)** The phosphorylation of Akt at tyrosine site 308 in liver tissue of mice. **P<0.01 vs chow, ^##^*P*<0.01, ^#^*P*<0.05 vs HFD, ^&^*P*<0.05 *Fndc5^-/-^* vs WT, n = 6. NS, no significance.

Next, we investigated the influence of Fndc5 knockout on the improvement of NR against HFD-induced insulin resistance. ITT assay showed that NR significantly improved insulin sensitivity in WT mice but to a much lesser extent in *Fndc5^-/-^* mice (**Figure 3A**). NR reduced AUC from ~1600 to ~1000 (about 60%) in WT mice but only reduced AUC from ~1600 to ~1400 (about 85%) in *Fndc5^-/-^* mice (**Figure 3B**). GTT also demonstrated that the NR treatment effectively improved insulin sensitivity in WT mice but to a much lesser extent in *Fndc5^-/-^* mice (**Figure 3C**), which was supported by the AUC results (**Figure 3D**). The activation of insulin signaling in liver tissue was also measured. The tyrosine phosphorylation of insulin receptor substrate 1 (IRS-1, Tyr-612 site), an essential procedure for activated insulin signaling transduction, was greatly inhibited in HFD-fed mice (**Figure 3E**). NR administration partially rescued the IRS-1 tyrosine phosphorylation in WT mice but to a much lesser extent in *Fndc5^-/-^* mice (**Figure 3E**). The serine phosphorylation of IRS-1 (Ser-307 and Ser-636 sites), which counteracted IRS-1 tyrosine phosphorylation and contributed to insulin resistance 53, was enhanced by HFD but suppressed by NR in WT mice but not in *Fndc5^-/-^* mice (**Figure 3F**). Accordingly, HFD caused a remarkable decline of Akt tyrosine phosphorylation (Thr-308) in WT mice, which was abolished in *Fndc5^-/-^* mice (**Figure 3G**). These data point out that Deletion of Fndc5 diminishes the improvement of NR against HFD-induced insulin resistance.

### Fndc5 deletion blunts the therapeutic efficacy of NR on steatohepatitis in NAFLD mice

**Figure 4:**
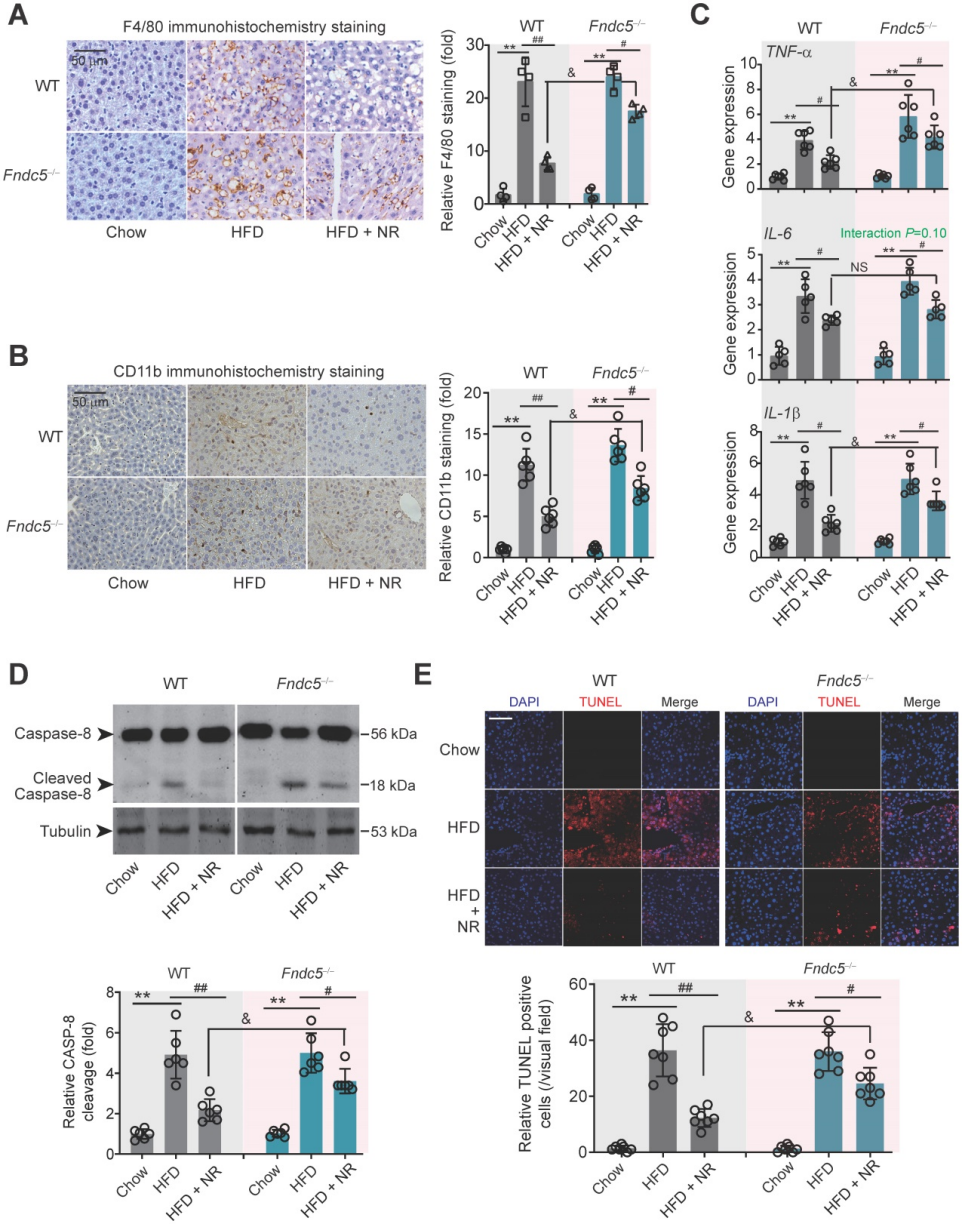
**Loss of Fndc5 counteracts the therapeutic efficacy of NR on HFD-induced hepatic inflammation and cell death. (A)** F4/80 immunohistochemistry staining in liver tissue of WT and *Fndc5^-/-^* mice. (**B**) CD11b immunohistochemistry staining in liver tissue of WT and *Fndc5^-/-^* mice. (**C**) Tissue TNF-α, IL-6 and IL-1β protein levels in liver tissue of WT and *Fndc5^-/-^* mice fed with HFD and NR. (**D**) The precursor and cleaved caspase-8 in liver tissue of WT and *Fndc5^-/-^* mice. (**E**) TUNEL staining in liver tissue of WT and *Fndc5^-/-^* mice. **P<0.01 vs chow, ^#^*P*<0.05, ^##^*P*<0.01 vs HFD, ^&^*P*<0.05 *Fndc5^-/-^* vs WT, n = 6. NS, no significance.

Immunohistochemical staining of F4/80, a marker of macrophage, showed that the intensity of F4/80-positive cells in liver of HFD-fed mice was much higher than that in mice fed chow, which was successfully attenuated by NR treatment in WT mice (**Figure 4A**). However, this therapeutic action of NR was blunted in *Fndc5^-/-^* mice (**Figure 4A**). Moreover, immunohistochemical staining of CD-11b, another molecular index for monocytes, demonstrated that NR significantly inhibited monocyte infiltration in liver tissues in WT mice but to a much lesser extent in *Fndc5^-/-^* mice (**Figure 4B**). We measured mRNA levels of three pro-inflammatory factors (tumor necrosis factor α [TNF-α], IL-6 and IL-1β) in liver tissues. NR significantly reduced HFD-induced TNF-α, IL-6 and IL-1β mRNA levels in WT mice but to a lesser extent in *Fndc5^-/-^* mice (**Figure 4C**). Immunoblotting of Caspase-8, an essential modulator of liver inflammation by initiating hepatocytes death 54, showed that NR treatment successfully suppressed HFD-induced Caspase-8 cleavage, while this action of was significantly weakened in *Fndc5^-/-^* mice (**Figure 4D**). In line with this, NR treatment substantially inhibited the HFD-induced increase of terminal dexynucleotidyl transferase (TdT)-mediated dUTP nick end labeling (TUNEL)-positive cell number in WT mice but to a much lesser extent in *Fndc5^-/-^* mice (**Figure 4E**). These data indicate that deletion of Fndc5 blunts therapeutic efficacy of NR on HFD-induced liver inflammation.

### Fndc5 deletion counteracts the therapeutic efficacy of NR on liver injury in NAFLD mice

**Figure 5:**
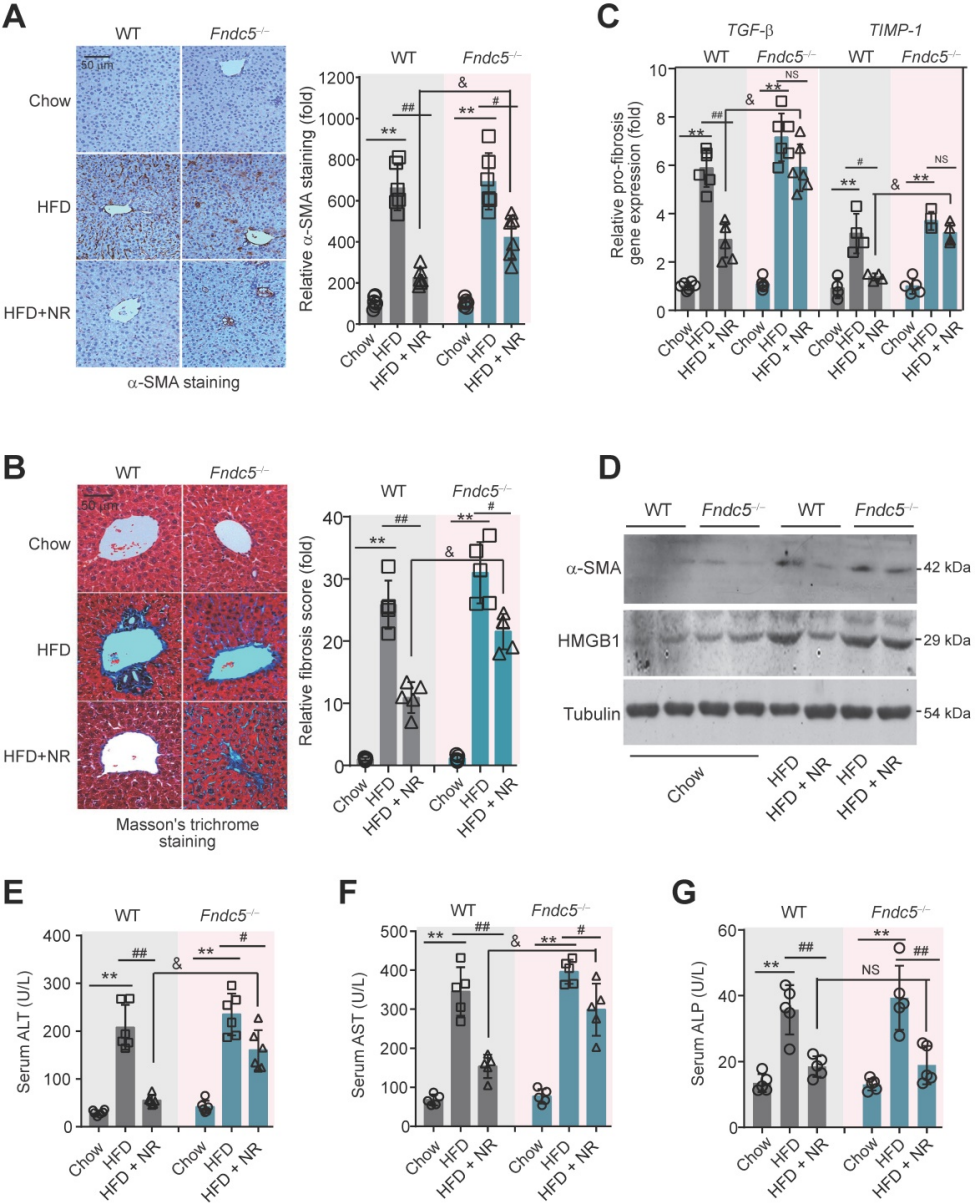
** Loss of Fndc5 counteracts the therapeutic efficacy of NR on HFD-induced liver fibrosis and injury. (A)** α-SMA staining in liver tissue of WT and *Fndc5^-/-^* mice. **(B)** Masson's staining in liver tissue of WT and *Fndc5^-/-^* mice. **(C)** qPCR analysis showing the *TGF-β* and *TIMP-1* mRNA in liver tissue of WT and *Fndc5^-/-^* mice. **(D)** Immunoblotting analysis showing the HMGB-1 protein expression in liver tissue of WT and *Fndc5^-/-^* mice. **(E-G)** Plasma activities of AST **(E)**, ALT **(F)** and ALP **(G)** in WT and *Fndc5^-/-^* mice. **P<0.01 vs chow, ^#^*P*<0.05, ^##^*P*<0.01 vs HFD, ^&^*P*<0.05 *Fndc5^-/-^* vs WT, n = 6. NS, no significance.

To further assess the role of Fndc5/irisin in therapeutic effects of NR, liver fibrosis was determined. The intensity of α-smooth muscle actin (α-SMA) immunohistochemical staining in liver tissue was obviously enhanced in HFD-fed mice in both WT and *Fndc5^-/-^* mice, suggesting that HFD induced liver fibrosis in these mice (**Figure 5A**). Masson's trichrome staining, a specific staining for the detection of collagen fibres, also demonstrated that NR treatment greatly reduced the HFD-induced collagen contents in livers of WT mice, and this action was much reduced in *Fndc5^-/-^* mice (**Figure 5B**). Quantitative PCR analysis of transforming growth factor-β (TGF-β) and tissue inhibitor of metalloproteinase-1 (TIMP-1), two well-established molecular maker of liver fibrosis 31, also showed that NR treatment decreased HFD-induced hepatic TGF-β and TIMP-1 mRNA expression in WT mice but not in *Fndc5^-/-^* mice (**Figure 5C**). The α-SMA and high-mobility group box-1 (HMGB-1), another important driver of liver fibrosis 55, were significantly increased by HFD in WT and *Fndc5^-/-^* mice liver tissues (**Figure 5D**). NR administration attenuated the upregulation of α-SMA and HMGB-1 in WT mice liver and to a much lesser extent in *Fndc5^-/-^* mice liver (**Figure 5D**). In addition, the inhibitory actions of NR on HFD-induced increases in plasma levels of alanine aminotransferase (ALT) and aspartate aminotransferase (AST) were significantly blunted in *Fndc5^-/-^* mice e (**Figure 5E-F**). However, NR reduced alkaline phosphatase (ALP) in WT mice and *Fndc5^-/-^* mice liver to a similar extent (**Figure 5G**).

### Fndc5 deletion retards the beneficial effects of NR on genes involved in mitochondrial biogenesis and mitophagy in NAFLD mice

**Figure 6:**
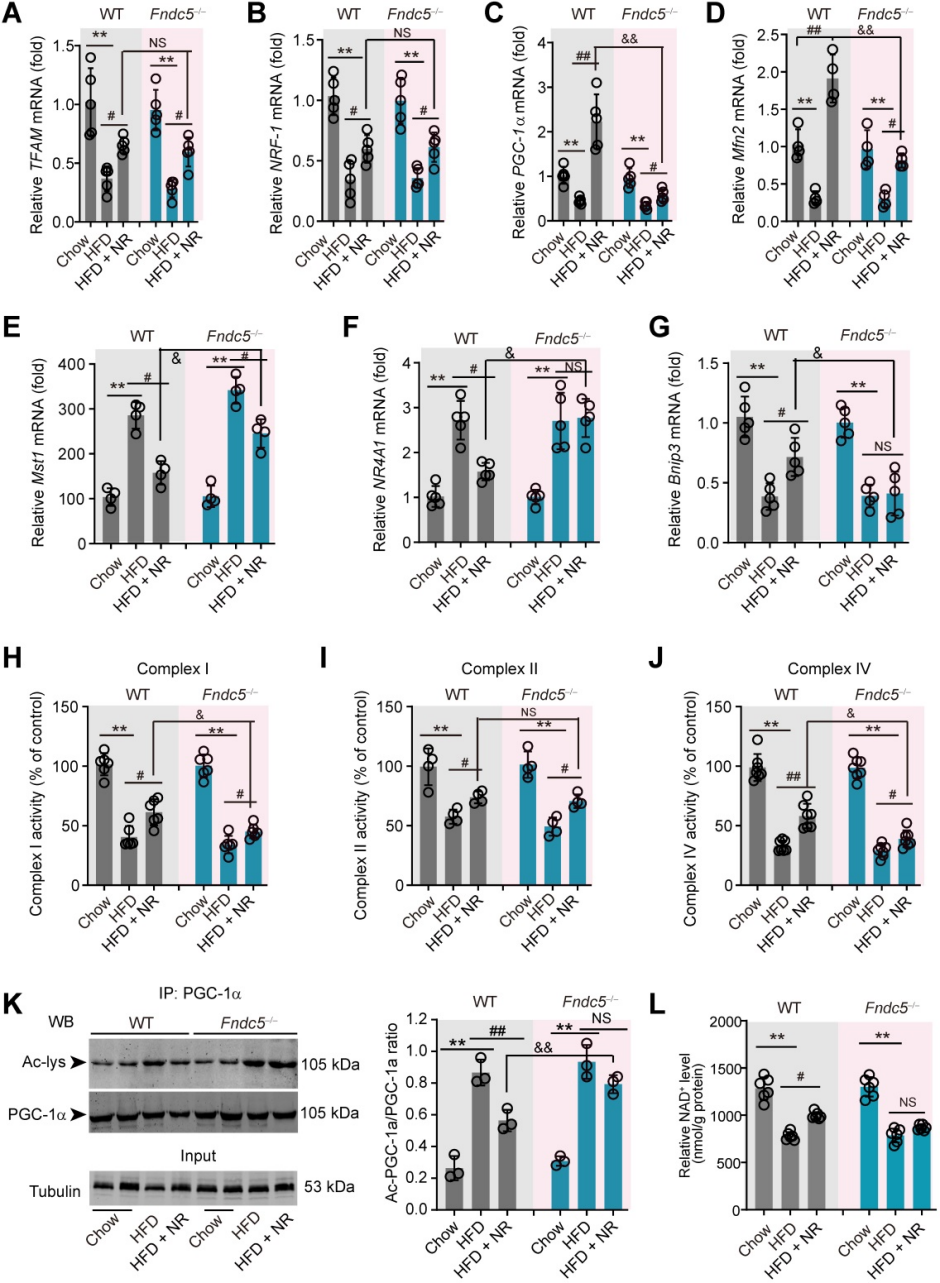
**Loss of Fndc5 revokes the beneficial effects of NR on genes involved in mitochondrial biogenesis and mitophagy in NAFLD mice.** (**A-G**) The mRNA expression of *TFMA* **(A)**, *NRF-1* **(B)**, *PGC-1α* **(C)**, *Mfn2* **(D)**, *Mst1* **(E)**, *NR4A1* **(F)** and *Bnip4* **(G)** in liver tissue of WT and FNDC5^-/-^ mice was determined using quantitative PCR analysis. **(H-J)** Mitochondria were isolated and the activities of Complex I, II and IV in mitochondrial extracts were determined. **(K)** The acetylation of PGC-1α in liver tissue of WT and FNDC5^-/-^ mice was measured. The liver samples were immunoprecipitated with a monoclonal against PGC-1α and then probed by an antibody against acetylated-lysine with immunoblotting. **(L)** The NAD^+^ levels in liver tissue of WT and FNDC5^-/-^ mice. *P<0.05 vs chow; ^#^*P*<0.05,^ ##^*P*<0.01 vs HFD; ^&^*P*<0.01 *Fndc^-/-^* vs WT, n = 6. NS, no significance.

Since mitochondrial dysfunction precedes insulin resistance and steatosis 56, we measured the expression of genes involved in mitochondrial biogenesis and mitophagy in WT and *Fndc5^-/-^* mice. Three mitochondrial biogenesis genes (*transcription factor A, mitochondrial* [*TFAM], nuclear factor erythroid-derived 2-related factor 1 [NRF-1]* and *peroxisome proliferator-activated receptor gamma coactivator 1-α [PGC-1α])* were downregulated by HFD and reversed by NR treatment (**Figure 6A-C**). Interestingly, Fndc5 knockout only affected the action of NR on* PGC-1α* (**Figure 6C**) but not *TFAM* and *NRF-1* (**Figure 6A-B**). The mitofusin-2 (*Mfn2*), a mitochondrial membrane protein that was critical for sustaining mitochondrial DNA stability 57, was also reduced in HFD-fed mice liver but substantially reversed by NR treatment in WT mice (**Figure 6D**). However, the increase of *Mfn2* mRNA expression by NR was compromised in* Fndc5^-/-^* mice (**Figure 6D**). Besides, we determined the mRNA expression of *Mst1*, *NR4A1* and* Bnip3*, three genes involved in mitophagy. HFD enhanced liver *Mst1* and *NR4A1*mRNA levels (**Figure 6E-F**), but decreased liver* Bnip3* mRNA level (**Figure 6G**). NR corrected the *Mst1* mRNA level in WT mice and to a lesser extent in* Fndc5^-/-^* mice (**Figure 6E**). Further, NR failed to restore the HFD-modulated *NR4A1* and *Bnip3* mRNA levels in* Fndc5^-/-^* mice (**Figure 6F-G**). HFD also caused significant decreases in activities of mitochondrial complex I, II and IV (**Figure 6H-J**). NR treatment partly enhanced activities of mitochondrial complex I, II and IV in WT mice and to a lesser extent in* Fndc5^-/-^* mice (**Figure 6H-J**). We also detected the deacetylation of mitochondrial PGC-1α, which is tightly linked to the limited PGC-1α transcriptional ability 58. In line with previous studies 59, HFD increased PGC-1α acetylation in the liver (**Figure 6K**). NR pronouncedly reduced the acetylation of PGC-1α in WT mice but not in *Fndc5^-/-^* mice e (**Figure 6K**). Accordingly, NR treatment enhanced the hepatic NAD^+^ levels in WT mice and, to a lesser extent in *Fndc5^-/-^* mice (**Figure 6L**). These data suggests that deletion of Fndc5 revokes the beneficial effects of NR on mitochondrial biogenesis and integrity.

### Treatment of recombinant Fndc5/irisin reverses hepatic steatosis and steatohepatitis in mice

**Figure 7:**
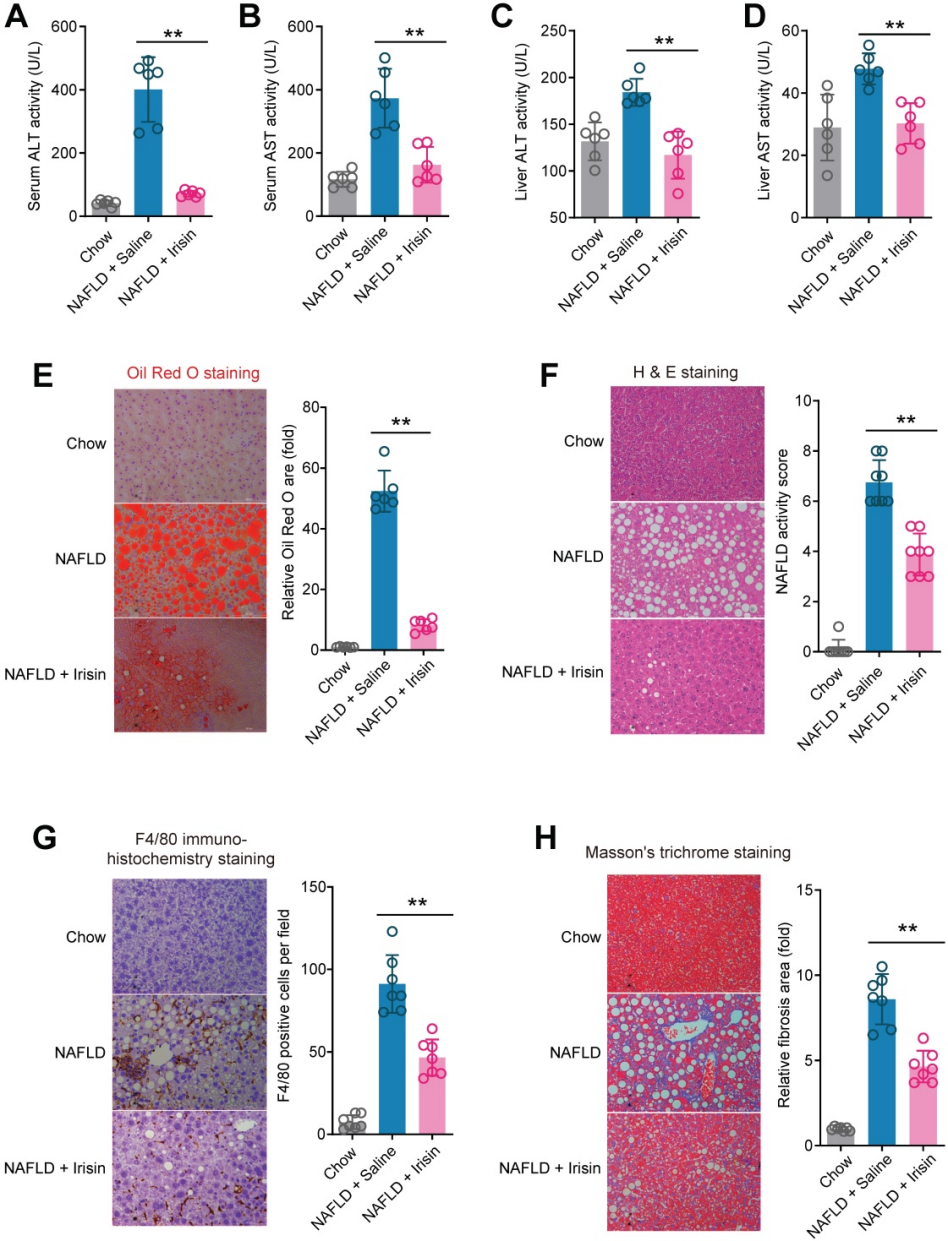
** Chronic infusion of recombinant irisin alleviates NAFLD in mice. (A-B)** Serum activities of ALT **(A)** and AST **(B)** in mice infused with recombinant irisin or saline water (vehicle) via Alzet osmotic minipump (5 nmol/kg body weight per day). **(C-D)** Liver tissue activities of ALT **(C)** and AST **(D)** in mice infused with recombinant irisin or saline water (vehicle) via Alzet osmotic minipump. **(E)** Representative images and quantitative analysis of lipid accumulation in liver tissue by Oil Red O staining. **(F)** Representative images and quantitative analysis of NAFLD severity in liver tissue by H & E staining. **(G)** Representative images and quantitative analysis of macrophage infiltration in liver tissue by F4/80 immunohistochemistry staining. **(H)** Representative images and quantitative analysis of fibrosis in liver tissue by Masson's trichrome staining. **P<0.01 vs NAFLD + Saline, n = 6-8 per group.

To answer whether direct Fndc5/irisin administration can reverse NAFLD, we treated the MCD-induced NAFLD mice with infusion of recombinant irisin via Alzet osmotic minipump. As shown in **Figure 7A-B**, one-week treatment of irisin successfully attenuated serum ALT and AST activities. This liver protection was confirmed by the results from liver ALT and AST activities (**Figure 7C-D**). Oil Red O staining showed that the lipid accumulation in liver was largely inhibited by irisin infusion (**Figure 7E**). H & E staining also demonstrated that the NAFLD activity score was inhibited by irisin treatment (**Figure 7F**). To assess the macrophage activation, we performed F4/80 immunohistochemistry staining and found irisin treatment significantly reduced F4/80^+^ macrophages infiltration (**Figure 7G**). At last, irisin treatment significantly reversed liver fibrosis in Masson's trichrome staining (**Figure 7H**). These data indicate that recombinant irisin is able to reverse hepatic steatosis and steatohepatitis.

### NR inhibits Fndc5 ubiquitination and stabilizes Fndc5 under lipid stress

**Figure 8:**
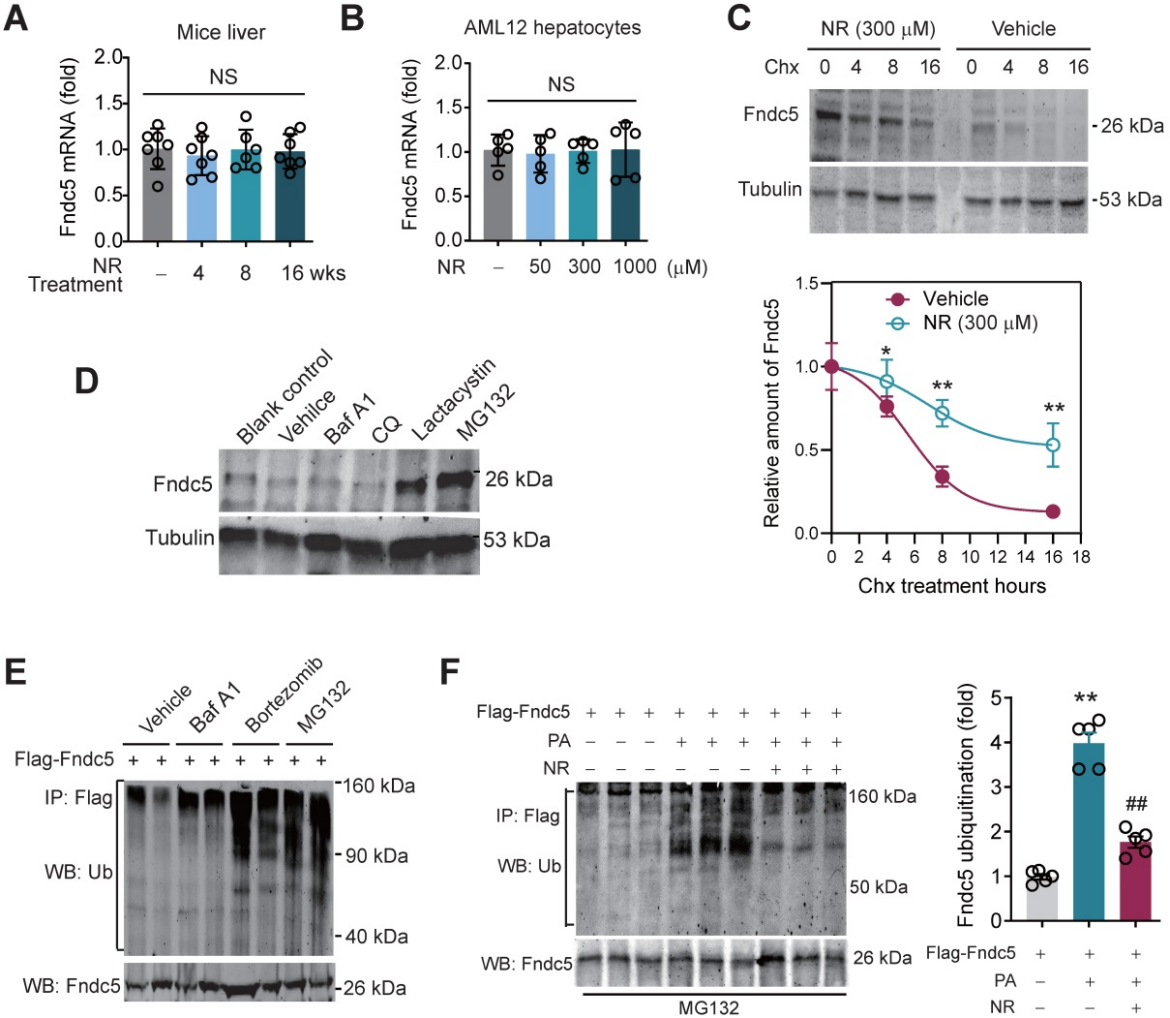
** NR inhibits ubiquitination of Fndc5 to stabilize Fndc5. (A-B)** *Fndc5* mRNA expression in mouse liver tissue **(A)** and AML12 hepatocytes **(B)** upon NR administration. n = 5-7. NS, no significance. **(C)** Evaluation of Fndc5 protein degradation and stability under vehicle control (saline) or NR treatment (300 µM) using cycloheximide (Chx, 3 µM) incubation in AML12 hepatocytes. *P<0.05, **P<0.01 vs control AML12 cells. n = 4. **(D)** Influence of lactacystin, MG132, bafilomycin A1 (Baf A1) and chloroquine (CQ) on Fndc5 protein expression. Tubulin was used as a loading control. **(E)** Effects of proteasome inhibitors (bortezomib and MG132) and autophagy inhibitor Baf-A1 on Fndc5 ubiquitination in cultured AML12 hepatocytes. The cell extracts were immunoprecipitated with anti-flag antibody and then probed by anti-ubiquitin and anti-Fndc5 antibodies. **(F)** Ubiquitination of Fndc5 under palmitic acid (PA) or NR. MG132 was added to block ubiquitin-proteasome degradation system. The cell extracts were immunoprecipitated with an anti-flag antibody and then probed by anti-ubiquitin and anti-Fndc5 antibodies. **P<0.01 vs without PA. ^##^*P*<0.01 vs PA. n = 4. IP, immunoprecipitation. WB, western blotting.

We next explored how NR stimulated Fndc5 protein expression. Intriguingly, NR treatment for 4~16 weeks did not change liver Fndc5 mRNA level in mice (**Figure 8A**). Moreover, NR administration (50-1000 µM) in cultured AML12 hepatocyte cell line for 48 hours did not alter Fndc5 mRNA level (**Figure 8B**). This discrepancy suggests a likelihood of posttranslational regulation of Fndc5. Hence, we next examined whether NR affects Fndc5 protein stability by evaluating the degradation curve of Fndc5 via blocking protein synthesis with cycloheximide (Chx). As shown in **Figure 8C**, the half-life of Fndc5 protein in hepatocytes was about 6 hours and became undetectable after 16 hours. In NR-treated cells, the Fndc5 protein half-life was much longer than control cells (> 16 hours). Furthermore, treatment with proteasome inhibitor lactacystin or MG132 dramatically increased Fndc5 levels in AML2 cells (**Figure 8D**). In contrast, bafilomycin A1 (Baf-A1) or chloroquine, two autophagy-lysosome inhibitors, failed to increase Fndc5 protein level (**Figure 8D**). These results suggest that NR increases Fndc5 protein stability by inhibiting its degradation via ubiquitin-proteasome rather than autophagy-lysosome system.

Hence, we studied whether Fndc5 is ubiquitinated and its ubiquitination can be modulated by lipid stress and NR. Treatment with proteasome inhibitors bortezomib and MG132, but not autophagy-lysosome inhibitor Baf-A1, significantly enhanced Fndc5 ubiquitination in hepatocytes (**Figure 8E**). Notably, in an *in vitro* NAFLD model induced by palmitic acid (PA) in AML12 hepatocyte cell line, the ubiquitination of Fndc5 was substantially increased under MG132 (the 2^nd^ lane from right, **Figure 8F**). NR supplement partially inhibited the increment of Fndc5 ubiquitination by PA (the 1^st^ lane from right, **Figure 8F**). In line with these results, the Fndc5 protein expression in cell lysates was reduced by PA but reversed by NR (the lowest gel, **Figure 8F**).

### NAD^+^-dependent SIRT2 promotes Fndc5 deacetylation and deubiquitination

**Figure 9:**
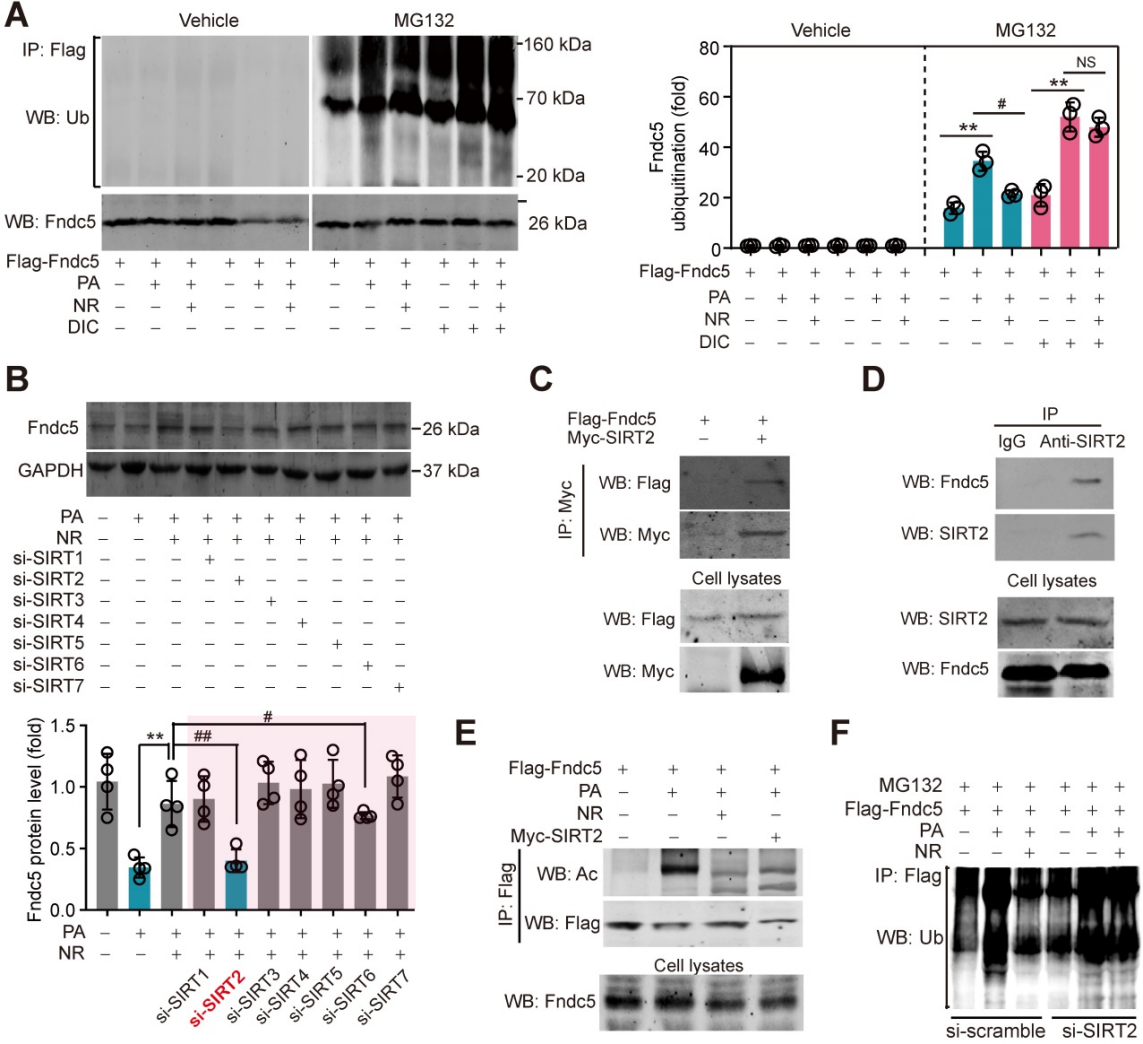
** SIRT2 interacts with Fndc5 to promote its deacetylation and deubiquitination. (A)** Inhibition of deacetylases by DIC abolishes the action of NR on Fndc5 deubiquitination in HepG2 cells. DIC, deacetylation inhibition cocktail (100×). The extracts of cells treated as indicated were immunoprecipitated with anti-flag antibody and then probed by anti-ubiquitin and anti-Fndc5 antibodies. **P<0.01 PA vs veh, ^#^*P*<0.05 NR vs PA; n = 3. NS, no significance. **(B)** Effects of siRNA-mediated SIRT1-7 knockdown on Fndc5 protein expression in HepG2 cells under PA and NR treatment. **P<0.01 NR vs PA; ^#^*P*<0.05,^ ##^*P*<0.01 vs PA + NR; n = 3. NS, no significance. **(C)** Flag-Fndc5 and myc-SIRT2 were co-transfected into HepG2 cells to detect the interaction between them using co-immunoprecipitation assay. **(D)** The interaction between endogenous Fndc5 and SIRT2 was detected using immunoprecipitation and Western blotting assay. Normal IgG was used as a normal control. **(E)** The influences of NR treatment or SIRT2 overexpression on Fndc5 acetylation were detected. The Flag-Fndc5-transfected cells treated as indicated were lysed and immunoprecipitated with an anti-flag antibody, and then probed by an anti-Ac-lys antibody. (**F**) The ubiquitination of Fndc5 in control and SIRT2-knockdown cells.

NAD^+^-boosting molecule elevates intracellular NAD^+^ pool, which is the essential for deacetylases activities 60. To investigate whether deacetylases participate in the regulation of Fndc5 ubiquitination, we treated hepatocytes with PA and NR in the absence and presence of deacetylation inhibition cocktail (DIC). NR attenuated PA-induced Fndc5 ubiquitination without DIC; however, this action was totally abolished in the presence of DIC (**Figure 9A**). Thus, we turned to screen which NAD^+^-dependent deacetylase is involved in the regulation of NR on Fndc5 using siRNA-mediated knockdown of SIRT1-SIRT7 (**Figure S3A**). Only knockdown of SIRT2 (**Figure S3B**) remarkably inhibited the rescue effect of NR on PA-induced Fndc5 downregulation (**Figure 9B**). We noted that knockdown of SIRT6 also partly inhibited the action of NR; however, the degree of SIRT2 knockdown-resulted in inhibition on Fndc5 was much greater than that of SIRT6 knockdown (**Figure 9B**). These suggest that SIRT2 may be a key link between NR and Fndc5. To explore whether SIRT2 directly interacts with Fndc5, we transfected Flag-tagged Fndc5 and Myc-tagged SIRT2 into HepG2 cells and performed co-immunoprecipitation assay. SIRT2 indeed interacted with Fndc5 in HepG2 cells (**Figure 9C**). The interaction between endogenous SIRT2 and Fndc5 was also confirmed in HepG2 cells (**Figure 9D**). The physiological correlation between SIRT2 and Fndc5 upon lipid stress was further evaluated. NR treatment successfully reduced PA-induced Fndc5 acetylation (**Figure 9E**). Overexpression of SIRT2 also produced a similar effect against PA-induced Fndc5 acetylation (**Figure 9E**). Furthermore, NR displayed a potent inhibition on PA-induced Fndc5 ubiquitination in control HepG2 cells but not in SIRT2-depleted HepG2 cells (**Figure 9F**), which suggests SIRT2 is essential for the effect of NR on Fndc5 deubiquitination. Together, these results showed that the NAD^+^ drives the deacetylase SIRT2 to promote Fndc5 deacetylation and deubiquitination.

### K127/131 and K185/187/189 sites, but not K177 site of Fndc5, may contribute to the SIRT2-dependent deacetylation and deubiquitination on Fndc5

**Figure 10:**
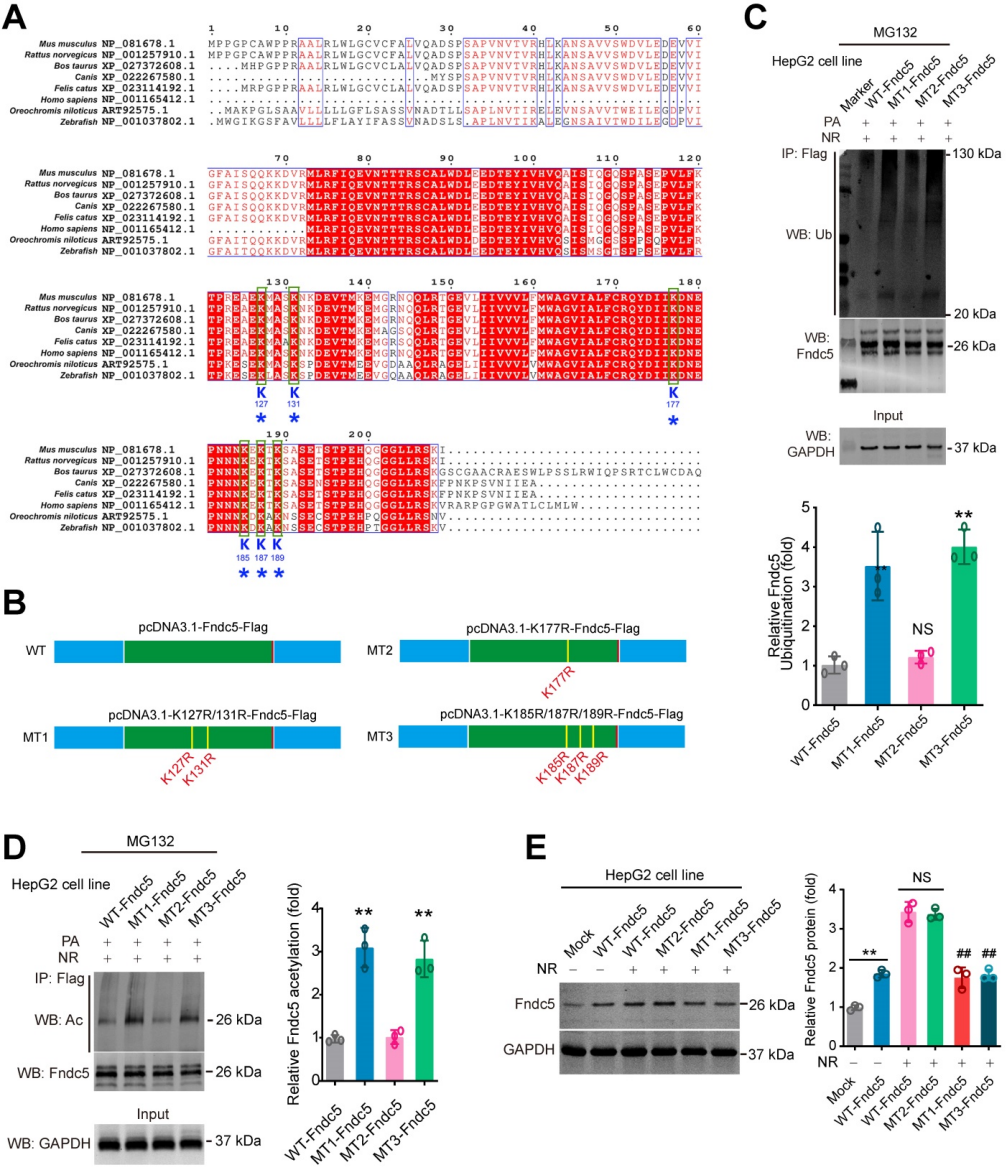
** Identification of key lysine sites for SIRT2-mediated Fndc5 deubiquitination and deacetylation. (A)** Comparison of Fndc5 protein sequences among mouse, rat, cattle, dog, cat, oreochromis niloticus, zebrafish and human species. The identical sites were highlighted by red and the conservative lysine sites were indicated by asterisks. **(B)** Plasmids carrying wild-type (WT) and mutant Fndc5 (MT) were generated. The K127 and K131 sites in MT1-Fndc5, K177 site in MT2-Fndc5 and K185, K187 and K189 sites in MT3-Fndc5 were mutated into arginine (R). **(C)** WT, MT1-Fndc5, MT2-Fndc5 and MT3-Fndc5 were transfected into HepG2 cell line respectively and then treated with PA and NR. To monitor the ubiquitination of these Fndc5 proteins, the cells were lysed and the extracts were immunoprecipitated by anti-Flag antibody followed by immunoblotting with anti-Ubiquitin. **P<0.01 vs WT-Fndc5. N = 3. **(D)** Cells were transfected and treated as in **(C)** and the cell extracts were immunoprecipitated by anti-Flag antibody followed by immunoblotting with anti-Acetylated-lysine (anti-Ac) to monitor the acetylation of Fndc5 proteins. **P<0.01 vs WT Fndc5. N = 3. **(E)** The Fndc5 protein expression in HepG2 cells transfected with plasmids carrying WT and mutant Fndc5 was determined. **P<0.01 vs Mock. ^##^*P*<0.01 vs WT-Fndc5. N = 3.

To explore which sites in Fndc5 may be critical for its deacetylation and deubiquitination by SIRT2, we compared the protein sequences of Fndc5 among a number of species, including mouse, rat, cattle, dog, cat, oreochromis niloticus, zebrafish and human. We found that K127/131, K177 and K185/187/189 lysine sites were conservative among these species (**Figure 10A**). Thus, we constructed three mutated Fndc5 with K127/131R (mutant 1, MT1-Fndc5), K177R (mutant 2, MT2-Fndc5) and K185/187/189R (mutant 3, MT3-Fndc5) with Flag-tags (**Figure 10B**). They were transfected into HepG2 cell line as well as the Flag-tagged wild-type Fndc5 (WT). The cells were stimulated by PA (an* in vitro* NAFLD model) and treated with NR. We found that in the cells transfected with MT1-Fndc5 and MT3-Fndc5, the ubiquitination (**Figure 10C**) and acetylation (**Figure 10D**) of Fndc5 were significantly higher than WT-Fndc5 or MT2-Fndc5 in the presence of PA and NR (**Figure 10C-D**), suggesting mutation of K127/131 and K185/187/189 sites might hamper the regulation of Fndc5 ubiquitination and acetylation by NR. Immunoblotting analysis showed that transfection of WT-Fndc5 or MT2-Fndc5 into HepG2 cells produced obvious overexpression of Fndc5 protein, whereas transfection of MT1-Fndc5 or MT3-Fndc5 did not induce such phenotype (**Figure 10E**), implying that mutation of K127/131 and K185/187/189 sites may affect Fndc5 protein structure. All together these results suggest that the K127/131 and K185/187/189, but not K177, may be the critical sites for SIRT2-dependent deacetylation and deubiquitination on Fndc5.

### Blockade of SIRT2 compromises the therapeutic action of NR against NAFLD

**Figure 11:**
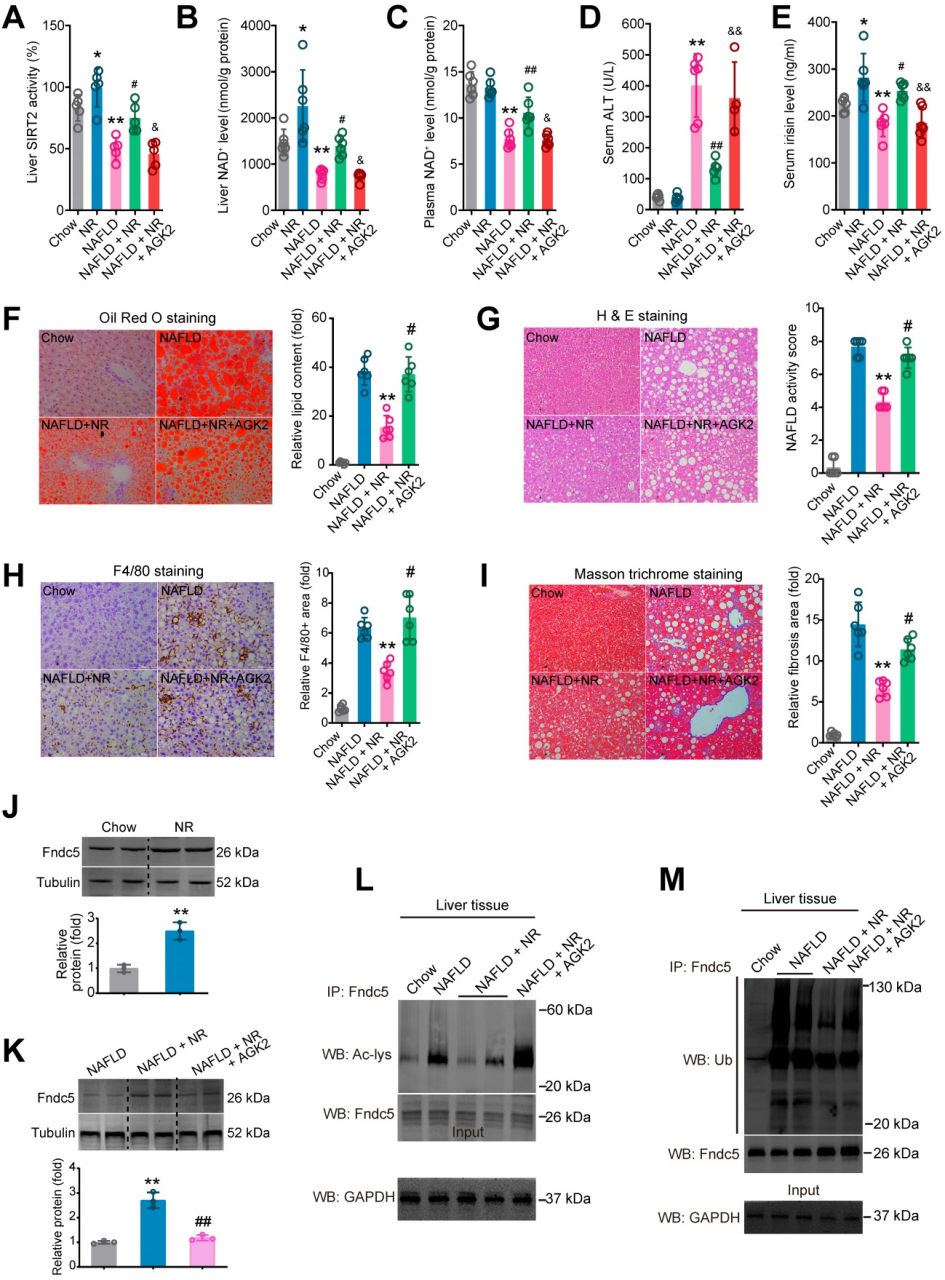
** Blockade of SIRT2 by AGK2 compromises the therapeutic action of NR against NAFLD. (A-E)** Liver SIRT2 activity **(A)**, liver NAD^+^ level **(B)**, plasma NAD^+^ level **(C)**, Serum ALT activity **(D)** and serum irisin level were determined in five groups of mice. NAFLD model was induced by MCD diet for 4 weeks. NR (400 mg/kg/day) and AGK2 (1 mg/kg/day) were injected intraperitoneally. *P < 0.05, **P < 0.01 vs Chow. ^#^*P* < 0.05, ^##^*P* < 0.01 vs NAFLD. ^&^*P* < 0.05, ^&&^*P* < 0.01 vs NAFLD + NR. N = 6 per group. **(F-I)** Representative images and quantitative analysis of lipid accumulation, NAFLD activity score, macrophage infiltration and liver fibrosis according to Oil Red O staining, H & E staining, F4/80 immunohistochemistry staining and Masson trichrome staining respectively. *P < 0.05, **P < 0.01 vs NAFLD. ^#^*P* < 0.05, ^##^*P* < 0.01 vs NAFLD + NR. N = 6 per group. **(J-K)** Fndc5 protein expression in mice under normal **(J)** or NAFLD **(K)** status. **(L-M)** The liver tissues were lysed and then immunoprecipitated by anti-Fndc5 antibody followed by immunoblotting with anti-Acetylated-lysine (anti-Ac, **L**) or anti-Ubiquitin **(M)** to monitor the acetylation or ubiquitination of endogenous Fndc5 in mice treated with NR or NR plus AGK2. AGK2 treatment significantly abolished the decreased acetylation and ubiquitination of Fndc5 by NR.

To ascertain that SIRT2 is required for deacetylation and deubiquitination on Fndc5 by NR, we used a SIRT2 selective inhibitor AGK2 in NAFLD model mice. NR treatment elevated SIRT2 activity in both Chow-fed and NAFLD model mice (**Figure 11A**). However, this elevation was abolished in the mice received co-treatment of NR and AGK2 (**Figure 11A**). Similar results were observed in liver NAD^+^ and plasma NAD^+^ levels (**Figure 11B-C**). AGK2 treatment also abrogated the inhibitory action of NR on serum ALT activity (**Figure 11D**) but not AST activity (**Figure S4A**). NR increased circulating irisin level in Chow-fed or NAFLD mice, which was compromised by AGK2 administration (**Figure 11E**). AGK2 supplement abolished the therapeutic action of NR on hepatic steatosis (**Figure 11F**), NAFLD activity score (**Figure 11G**) and F4/80^+^ macrophage infiltration (**Figure 11H**). Similar observation was noted in liver fibrosis, evidenced by Masson trichrome staining (**Figure 11I**), Sirus Red staining (**Figure S4B**) and α-SMA immunohistochemistry staining (**Figure S4C**). NR treatment did not alter Fndc5 mRNA level in mice (**Figure S4A**), but elevated Fndc5 protein level (**Figure 11J**). Notably, AGK2 inhibited the upregulation of Fndc5 protein by NR (**Figure 11K**). AGK2 significantly impaired the NR-induced deacetylation (**Figure 11L**) and deubiquitination (**Figure 11M**) of Fndc5 in liver tissues from these mice. These results indicate that blockade of SIRT2 impairs the therapeutic action of NR against NAFLD and regulation of Fndc5 deacetylation and deubiquitination by NR.

## Discussion

The NAD^+^-boosting therapy via 'NAD^+^-boosting molecules' such as NR and NMN has attracted considerable attention since it has shown great potential in various disorders including NAFLD. All NAD^+^ precursors (NR, NMN, niacin, nicotinamide and nicotinic acid mononucleotide) can effectively elevate NAD^+^ level; however, NR seems to be superior to NA and nicotinamide in elevating NAD^+^ content 22, and has been tested in human preclinical trials 23. Thus, we used NR as the NAD^+^-boosting agent in the present study. As an oral agent, NR unavoidably causes plenty of biological functions outside liver 10,20. Previous studies reported that blood irisin levels were reduced in NAFLD 61 but triggered by exercise and cold 35,36,39. Moreover, serum irisin concentrations were reported to be inversely associated with the triglyceride contents in the liver in obese adults 61. By screening the circulating metabolism-related factors found that the exerkine irisin, the cleaved product of Fndc5, was enhanced by NR administration. These imply a link between irisin and NAD^+^-boosting therapy.

Intrigued by these findings, we further asked whether the stimulated Fndc5/irisin is critical for the anti-NAFLD activity of NR. Due to its wide distribution and presence in both extracellular and intracellular spaces, Fndc5/irisin may function via multiple mechanisms. As an extracellular cytokine, irisin regulates hepatic ischemia-reperfusion injury 62, cholesterol synthesis 43 and gluconeogenesis 63. Two independent groups showed that the rs3480 polymorphism in Fndc5 3' untranslated region was associated with hepatic steatosis and fibrogenesis in human NAFLD patients 64,65. Blood irisin concentration was reduced in patients with NAFLD/NASH 66 and obese adults 61. Our work showed that loss of Fndc5 globally substantially impaired the protection of NR against NAFLD. These results point out that the exercise-linked hormone Fndc5/irisin is a pivotal mediator of NAD^+^-boosting therapy against NAFLD. This previously-unknown mechanism is likely to explain the enhanced NAD^+^ pool during exercise and the therapeutic activity of exercise on NAFLD. NR supplement and exercise exhibited comparable simulative effects on plasma irisin in a small sample size of volunteers, suggesting Fndc5/irisin may be a link between NAD^+^ and physical exercise. More importantly, direct treatment of irisin by minipump successfully alleviated NAFLD in mice. Thus, the speculation that NAD^+^-boosting molecules can mimics physical exercise, at least partly, should be investigated by more experimental works and replicated with larger samples in human. It is noteworthy that some previous studies have reported HFD feeding even up to 9 months was hard to induce fibrosis 67,68. We used C57BL76J mice in the present study, which have a mutation in the NADPH-producing enzyme nicotinamide nucleotide transhydrogenase (NNT), which might cause mitochondrial dysfunction and increase susceptibility to metabolism disorders 69,70. Furthermore, NNT mutation may render these mice more sensitive to the protection induced by NR supplement since NR directly promotes NAD^+^ production. As both NNT and NAMPT lie in the NAD^+^ generation signaling, the complex crosstalk between these two enzymes may be an interesting issue for future investigations.

Post translational modification (PTM) is a major means to modulate protein activity. We for the first time revealed that upon lipid stress, acetylation and ubiquitination of Fndc5 were significantly enhanced, which promoted Fndc5 degradation and thus reduced Fndc5 protein level. The reduced Fndc5 protein would naturally lend to a decrease of irisin release into circulating blood and intercellular space. These events were counteracted by NR via promoting Fndc5 deacetylation and deubiquitination. Finally, we confirmed the interaction between Fndc5 and SIRT2, and found depletion of SIRT2 blocked the action of NR on Fndc5 ubiquitination. This indicates SIRT2 is required for the regulation of Fndc5 protein stability. Our work is the first demonstration of Fndc5 deacetylation/ubiquitination and stability, which is supported by several lines of evidence. First, the production of irisin from full-length Fndc5 via proteolytically cleavage is a kind of PTM 35. Physiological doses of leptin had no effect on Fndc5 mRNA level in C_2_C_12_ myotubes but increased circulating irisin concentrations in rats by affecting its proteolytically cleavage 71. In another study, Fndc5/irisin was detected in ~40, 58, and 75 kDa in immunoblotting, which were confirmed to contain Fndc5 peptide by mass spectrometry 37. Second, the fibronectin type III domain, which is the major structure of Fndc5 protein, is a small autonomous folding unit functioning as a scaffold for novel binding proteins 72. This suggests that Fndc5 may interact with other proteins during various biological functions. Moreover, it should be noted that two recent independent groups have reported that the stability of Fndc5 mRNA was regulated by its rs3480 polymorphism in 3' untranslated region 64,65, suggesting that transcriptional regulation of Fndc5 such as microRNA- or long non-coding RNA-mediated RNA processing, may be also involved in the Fndc5 regulation. As we demonstrate a new type of epigenetic regulatory mechanism of Fndc5 in this study, the homeostasis of the exerkine Fndc5 in cell is still an open question.

Our results suggest that SIRT2 may be the deacetylase of Fdnc5. Mammalian sirtuins, the major mediator of NAD^+^ biological functions, have seven homologs (SIRT1-7). They function as class III histone deacetylases (HDACs) by hydrolyzing one NAD^+^ and releasing nicotinamide with different deacetylase activities and distinct subcellular localizations. Unlike other sirtuins, SIRT2 is the only sirtuin that resides predominantly in the cytoplasm 73. It was previously reported that NR reverted NAFLD by inducing SIRT1 and SIRT3-dependent intracellular mitochondrial unfolded protein response in hepatocytes 29. Nevertheless, it seemed that SIRT1 and SIRT3 may not the only involved sirtuins because we previously found overexpression of SIRT1 in liver seemed to fail to fully mimic the protection of NR 31. By contrast, our results unraveled that SIRT2 is an essential factor for NAD^+^-mediated biological functions in NAFLD by controlling Fndc5 deacetylation/ubiquitination and protein stability. Furthermore, we identified K127/131 and K185/187/189 sites of Fndc5 are critical for its deacetylation by SIRT2 and thereby the deubiquitination. During this interaction, SIRT2 may play key roles via their deacetylate activities. In line with our findings, Lantier* et al.* reported that SIRT2-KO mice exhibited reduced insulin-induced glucose uptake, impaired insulin resistance, increased body weight and dysfunctional mitochondrion biosynthesis upon high-fat diet 74. SIRT2 inhibitor AGK2 can blocked the anti‐NAFLD effect of silybin, a traditional Chinese medicines used as a hepatoprotective agent 75. This new regulatory pattern on exercise-linked hormone Fndc5/irisin by NAD^+^-boosting molecule implicate the potential relationship between NAD^+^-boosting therapy and physical exercise.

## Conclusions

**Figure 12:**
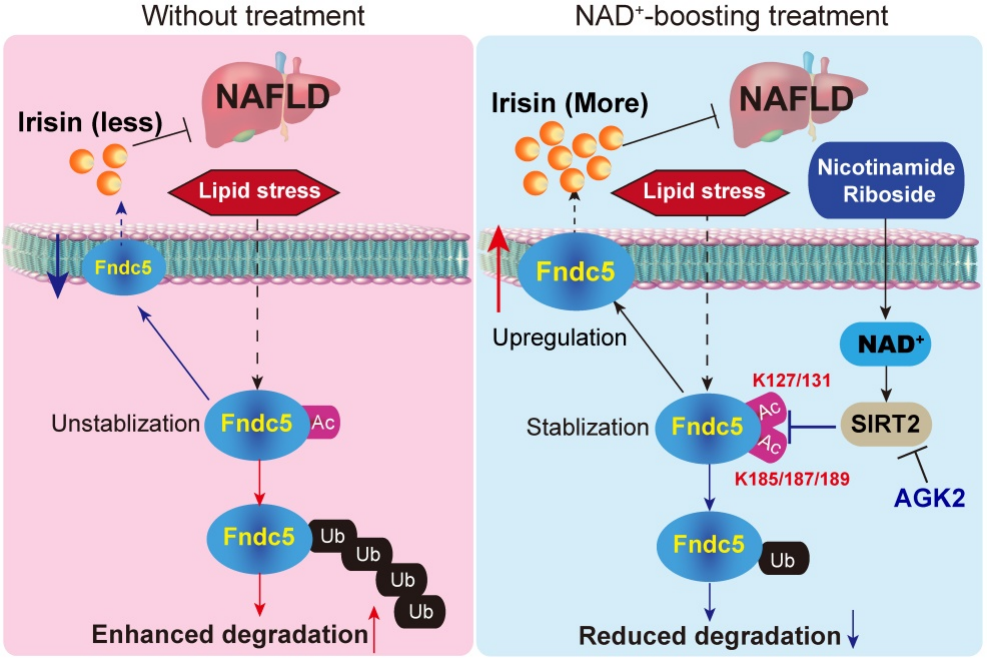
A proposed framework for the involvement of Fndc5/irisin in the protection of NR against NAFLD.

In conclusion, the present study indicates that NR protects against HFD-induced NAFLD by stimulating NAD^+^-dependent SIRT2 to promote Fndc5 deacetylation and deubiquitination, and then stabilizes Fndc5 (**Figure 12**). To our knowledge, this is the first study comprehensively describing the key roles of exerkine Fndc5/irisin in mediating therapeutic functions of NAD^+^-boosting therapy in metabolism disorders, further supporting the translational potential of NAD^+^-boosting molecules or irisin as exercise mimetics in clinical.

## Supplementary Material

Supplementary figures and tables.

## Abbreviations

ABCG1: ATP binding cassette subfamily G member 1
ACCα: acetyl-coenzyme carboxylase A
Acox1: acyl-CoA oxidase 1
CXCL10: C-X-C motif chemokine 10
ELISA: Enzyme-linked immunosorbent assay
Fndc5: fibronectin type III domain containing 5
GTT: glucose tolerance test
HFD: high-fat diet
IL-6: interleukin-6
ITT: insulin tolerance test
LDL-R: low density lipoprotein receptor
NAD^+^: nicotinamide adenine dinucleotide^+^
NAFLD: nonalcoholic fatty liver disease
NAMPT: nicotinamide phosphoribosyltransferase
NASH: nonalcoholic steatohepatitis
NMN: nicotinamide mononucleotide
NR: nicotinamide riboside
NRF-1: nuclear factor erythroid-derived 2-related factor 1
PGC-1α: peroxisome proliferator-activated receptor gamma coactivator 1-α
PPARα: peroxisome proliferator activated receptor-alpha
SREBP-2: sterol regulatory element binding transcription factor 2
TBST: Tris buffered saline with Tween 20
TFAM: transcription factor A, mitochondrial
TGF-β: transforming growth factor-β
TIMP-1: tissue inhibitor of metalloproteinase-1
TUNEL: terminal dexynucleotidyl transferase (TdT)-mediated dUTP nick end labeling
WT: wild-type

## References

[1] Cohen JC, Horton JD, Hobbs HH (2011). Human fatty liver disease: old questions and new insights. *Science*.

[2] Anstee QM, Targher G, Day CP (2013). Progression of NAFLD to diabetes mellitus, cardiovascular disease or cirrhosis. *Nat Rev Gastroenterol Hepatol*.

[3] Gaggini M, Morelli M, Buzzigoli E, DeFronzo RA, Bugianesi E, Gastaldelli A (2013). Non-alcoholic fatty liver disease (NAFLD) and its connection with insulin resistance, dyslipidemia, atherosclerosis and coronary heart disease. *Nutrients*.

[4] Yang Y, Sauve AA (2016). NAD(+) metabolism: Bioenergetics, signaling and manipulation for therapy. *Biochim Biophys Acta*.

[5] Revollo JR, Korner A, Mills KF, Satoh A, Wang T, Garten A (2007). Nampt/PBEF/Visfatin regulates insulin secretion in beta cells as a systemic NAD biosynthetic enzyme. *Cell Metab*.

[6] Bieganowski P, Brenner C (2004). Discoveries of nicotinamide riboside as a nutrient and conserved NRK genes establish a Preiss-Handler independent route to NAD+ in fungi and humans. *Cell*.

[7] Imai S, Guarente L (2014). NAD+ and sirtuins in aging and disease. *Trends Cell Biol*.

[8] Kim MY, Mauro S, Gevry N, Lis JT, Kraus WL (2004). NAD+-dependent modulation of chromatin structure and transcription by nucleosome binding properties of PARP-1. *Cell*.

[9] Camacho-Pereira J, Tarrago MG, Chini CCS, Nin V, Escande C, Warner GM (2016). CD38 Dictates Age-Related NAD Decline and Mitochondrial Dysfunction through an SIRT3-Dependent Mechanism. *Cell Metab*.

[10] Vannini N, Campos V, Girotra M, Trachsel V, Rojas-Sutterlin S, Tratwal J (2019). The NAD-Booster Nicotinamide Riboside Potently Stimulates Hematopoiesis through Increased Mitochondrial Clearance. *Cell Stem Cell*.

[11] Belenky P, Racette FG, Bogan KL, McClure JM, Smith JS, Brenner C (2007). Nicotinamide riboside promotes Sir2 silencing and extends lifespan via Nrk and Urh1/Pnp1/Meu1 pathways to NAD+. *Cell*.

[12] Zhang H, Ryu D, Wu Y, Gariani K, Wang X, Luan P (2016). NAD(+) repletion improves mitochondrial and stem cell function and enhances life span in mice. *Science*.

[13] Yoshida M, Satoh A, Lin JB, Mills KF, Sasaki Y, Rensing N (2019). Extracellular Vesicle-Contained eNAMPT Delays Aging and Extends Lifespan in Mice. *Cell Metab*.

[14] Canto C, Houtkooper RH, Pirinen E, Youn DY, Oosterveer MH, Cen Y (2012). The NAD(+) precursor nicotinamide riboside enhances oxidative metabolism and protects against high-fat diet-induced obesity. *Cell Metab*.

[15] Yoshino J, Mills KF, Yoon MJ, Imai S (2011). Nicotinamide mononucleotide, a key NAD(+) intermediate, treats the pathophysiology of diet- and age-induced diabetes in mice. *Cell Metab*.

[16] Wang P, Xu TY, Guan YF, Tian WW, Viollet B, Rui YC (2011). Nicotinamide phosphoribosyltransferase protects against ischemic stroke through SIRT1-dependent adenosine monophosphate-activated kinase pathway. *Ann Neurol*.

[17] Brown KD, Maqsood S, Huang JY, Pan Y, Harkcom W, Li W (2014). Activation of SIRT3 by the NAD(+) precursor nicotinamide riboside protects from noise-induced hearing loss. *Cell Metab*.

[18] Hasegawa K, Wakino S, Simic P, Sakamaki Y, Minakuchi H, Fujimura K (2013). Renal tubular Sirt1 attenuates diabetic albuminuria by epigenetically suppressing Claudin-1 overexpression in podocytes. *Nat Med*.

[19] Das A, Huang GX, Bonkowski MS, Longchamp A, Li C, Schultz MB (2019). Impairment of an Endothelial NAD(+)-H2S Signaling Network Is a Reversible Cause of Vascular Aging. *Cell*.

[20] Diguet N, Trammell SAJ, Tannous C, Deloux R, Piquereau J, Mougenot N (2018). Nicotinamide Riboside Preserves Cardiac Function in a Mouse Model of Dilated Cardiomyopathy. *Circulation*.

[21] Conze DB, Crespo-Barreto J, Kruger CL (2016). Safety assessment of nicotinamide riboside, a form of vitamin B3. *Hum Exp Toxicol*.

[22] Trammell SA, Schmidt MS, Weidemann BJ, Redpath P, Jaksch F, Dellinger RW (2016). Nicotinamide riboside is uniquely and orally bioavailable in mice and humans. *Nat Commun*.

[23] Martens CR, Denman BA, Mazzo MR, Armstrong ML, Reisdorph N, McQueen MB (2018). Chronic nicotinamide riboside supplementation is well-tolerated and elevates NAD(+) in healthy middle-aged and older adults. *Nat Commun*.

[24] Elhassan YS, Kluckova K, Fletcher RS, Schmidt MS, Garten A, Doig CL (2019). Nicotinamide Riboside Augments the Aged Human Skeletal Muscle NAD(+) Metabolome and Induces Transcriptomic and Anti-inflammatory Signatures. *Cell Rep*.

[25] Dollerup OL, Christensen B, Svart M, Schmidt MS, Sulek K, Ringgaard S (2018). A randomized placebo-controlled clinical trial of nicotinamide riboside in obese men: safety, insulin-sensitivity, and lipid-mobilizing effects. *Am J Clin Nutr*.

[26] Dollerup OL, Trammell SAJ, Hartmann B, Holst JJ, Christensen B, Moller N (2019). Effects of Nicotinamide Riboside on Endocrine Pancreatic Function and Incretin Hormones in Nondiabetic Men With Obesity. *J Clin Endocrinol Metab*.

[27] Dollerup OL, Chubanava S, Agerholm M, Sondergard SD, Altintas A, Moller AB (2020). Nicotinamide riboside does not alter mitochondrial respiration, content or morphology in skeletal muscle from obese and insulin-resistant men. *J Physiol*.

[28] Dall M, Trammell SAJ, Asping M, Hassing AS, Agerholm M, Vienberg SG (2019). Mitochondrial function in liver cells is resistant to perturbations in NAD(+) salvage capacity. *J Biol Chem*.

[29] Gariani K, Menzies KJ, Ryu D, Wegner CJ, Wang X, Ropelle ER (2016). Eliciting the mitochondrial unfolded protein response by nicotinamide adenine dinucleotide repletion reverses fatty liver disease in mice. *Hepatology*.

[30] Mukherjee S, Chellappa K, Moffitt A, Ndungu J, Dellinger RW, Davis JG (2017). Nicotinamide adenine dinucleotide biosynthesis promotes liver regeneration. *Hepatology*.

[31] Zhou CC, Yang X, Hua X, Liu J, Fan MB, Li GQ (2016). Hepatic NAD(+) deficiency as a therapeutic target for non-alcoholic fatty liver disease in ageing. *Br J Pharmacol*.

[32] Katsyuba E, Mottis A, Zietak M, De Franco F, van der Velpen V, Gariani K (2018). De novo NAD(+) synthesis enhances mitochondrial function and improves health. *Nature*.

[33] Vinel C, Lukjanenko L, Batut A, Deleruyelle S, Pradere JP, Le Gonidec S (2018). The exerkine apelin reverses age-associated sarcopenia. *Nat Med*.

[34] Choi SH, Bylykbashi E, Chatila ZK, Lee SW, Pulli B, Clemenson GD (2018). Combined adult neurogenesis and BDNF mimic exercise effects on cognition in an Alzheimer's mouse model. *Science*.

[35] Bostrom P, Wu J, Jedrychowski MP, Korde A, Ye L, Lo JC (2012). A PGC1-alpha-dependent myokine that drives brown-fat-like development of white fat and thermogenesis. *Nature*.

[36] Lee P, Linderman JD, Smith S, Brychta RJ, Wang J, Idelson C (2014). Irisin and FGF21 are cold-induced endocrine activators of brown fat function in humans. *Cell Metab*.

[37] Lourenco MV, Frozza RL, de Freitas GB, Zhang H, Kincheski GC, Ribeiro FC (2019). Exercise-linked FNDC5/irisin rescues synaptic plasticity and memory defects in Alzheimer's models. *Nat Med*.

[38] Chen K, Xu Z, Liu Y, Wang Z, Li Y, Xu X (2017). Irisin protects mitochondria function during pulmonary ischemia/reperfusion injury. *Sci Transl Med*.

[39] Kim H, Wrann CD, Jedrychowski M, Vidoni S, Kitase Y, Nagano K (2018). Irisin Mediates Effects on Bone and Fat via alphaV Integrin Receptors. *Cell*.

[40] Liu TY, Xiong XQ, Ren XS, Zhao MX, Shi CX, Wang JJ (2016). FNDC5 Alleviates Hepatosteatosis by Restoring AMPK/mTOR-Mediated Autophagy, Fatty Acid Oxidation, and Lipogenesis in Mice. *Diabetes*.

[41] Hu J, Wang H, Li X, Liu Y, Mi Y, Kong H (2020). Fibrinogen-like protein 2 aggravates nonalcoholic steatohepatitis via interaction with TLR4, eliciting inflammation in macrophages and inducing hepatic lipid metabolism disorder. *Theranostics*.

[42] Jin K, Liu Y, Shi Y, Zhang H, Sun Y, Zhangyuan G (2020). PTPROt aggravates inflammation by enhancing NF-kappaB activation in liver macrophages during nonalcoholic steatohepatitis. *Theranostics*.

[43] Tang H, Yu R, Liu S, Huwatibieke B, Li Z, Zhang W (2016). Irisin inhibits hepatic cholesterol synthesis via AMPK-SREBP2 signaling. *EBioMedicine*.

[44] Kim YY, Hur G, Lee SW, Lee SJ, Lee S, Kim SH (2020). AGK2 ameliorates mast cell-mediated allergic airway inflammation and fibrosis by inhibiting FcepsilonRI/TGF-beta signaling pathway. *Pharmacol Res*.

[45] Goldner J (1938). A modification of the masson trichrome technique for routine laboratory purposes. *Am J Pathol*.

[46] Buonfiglio D, Tchio C, Furigo I, Donato J Jr, Baba K, Cipolla-Neto J (2019). Removing melatonin receptor type 1 signaling leads to selective leptin resistance in the arcuate nucleus. *J Pineal Res*.

[47] Chen D, Mei Y, Kim N, Lan G, Gan CL, Fan F (2020). Melatonin directly binds and inhibits death-associated protein kinase 1 function in Alzheimer's disease. *J Pineal Res*.

[48] Hua X, Sun DY, Zhang WJ, Fu JT, Tong J, Sun SJ (2020). P7C3-A20 alleviates fatty liver by shaping gut microbiota and inducing FGF21/FGF1, via the AMP-activated protein kinase/CREB regulated transcription coactivator 2 pathway. *Br J Pharmacol*.

[49] Li DJ, Tong J, Zeng FY, Guo M, Li YH, Wang H (2019). Nicotinic ACh receptor alpha7 inhibits PDGF-induced migration of vascular smooth muscle cells by activating mitochondrial deacetylase sirtuin 3. *Br J Pharmacol*.

[50] Li DJ, Tong J, Li YH, Meng HB, Ji QX, Zhang GY (2019). Melatonin safeguards against fatty liver by antagonizing TRAFs-mediated ASK1 deubiquitination and stabilization in a beta-arrestin-1 dependent manner. *J Pineal Res*.

[51] Roca-Rivada A, Castelao C, Senin LL, Landrove MO, Baltar J, Belen Crujeiras A (2013). FNDC5/irisin is not only a myokine but also an adipokine. *PLoS One*.

[52] Aydin S, Kuloglu T, Aydin S, Kalayci M, Yilmaz M, Cakmak T (2014). A comprehensive immunohistochemical examination of the distribution of the fat-burning protein irisin in biological tissues. *Peptides*.

[53] Pederson TM, Kramer DL, Rondinone CM (2001). Serine/threonine phosphorylation of IRS-1 triggers its degradation: possible regulation by tyrosine phosphorylation. *Diabetes*.

[54] Hatting M, Zhao G, Schumacher F, Sellge G, Al Masaoudi M, Gabetaler N (2013). Hepatocyte caspase-8 is an essential modulator of steatohepatitis in rodents. *Hepatology*.

[55] Ge X, Arriazu E, Magdaleno F, Antoine DJ, Dela Cruz R, Theise N (2018). High Mobility Group Box-1 Drives Fibrosis Progression Signaling via the Receptor for Advanced Glycation End Products in Mice. *Hepatology*.

[56] Rector RS, Thyfault JP, Uptergrove GM, Morris EM, Naples SP, Borengasser SJ (2010). Mitochondrial dysfunction precedes insulin resistance and hepatic steatosis and contributes to the natural history of non-alcoholic fatty liver disease in an obese rodent model. *J Hepatol*.

[57] Chen H, Vermulst M, Wang YE, Chomyn A, Prolla TA, McCaffery JM (2010). Mitochondrial fusion is required for mtDNA stability in skeletal muscle and tolerance of mtDNA mutations. *Cell*.

[58] Rodgers JT, Lerin C, Haas W, Gygi SP, Spiegelman BM, Puigserver P (2005). Nutrient control of glucose homeostasis through a complex of PGC-1alpha and SIRT1. *Nature*.

[59] Minor RK, Baur JA, Gomes AP, Ward TM, Csiszar A, Mercken EM (2011). SRT1720 improves survival and healthspan of obese mice. *Sci Rep*.

[60] Rajman L, Chwalek K, Sinclair DA (2018). Therapeutic Potential of NAD-Boosting Molecules: The *In vivo* Evidence. *Cell Metab*.

[61] Zhang HJ, Zhang XF, Ma ZM, Pan LL, Chen Z, Han HW (2013). Irisin is inversely associated with intrahepatic triglyceride contents in obese adults. *J Hepatol*.

[62] Bi J, Zhang J, Ren Y, Du Z, Li Q, Wang Y (2019). Irisin alleviates liver ischemia-reperfusion injury by inhibiting excessive mitochondrial fission, promoting mitochondrial biogenesis and decreasing oxidative stress. *Redox Biol*.

[63] Liu TY, Shi CX, Gao R, Sun HJ, Xiong XQ, Ding L (2015). Irisin inhibits hepatic gluconeogenesis and increases glycogen synthesis via the PI3K/Akt pathway in type 2 diabetic mice and hepatocytes. *Clin Sci (Lond)*.

[64] Metwally M, Bayoumi A, Romero-Gomez M, Thabet K, John M, Adams LA (2019). A polymorphism in the Irisin-encoding gene (FNDC5) associates with hepatic steatosis by differential miRNA binding to the 3'UTR. *J Hepatol*.

[65] Petta S, Valenti L, Svegliati-Baroni G, Ruscica M, Pipitone RM, Dongiovanni P (2017). Fibronectin Type III Domain-Containing Protein 5 rs3480 A>G Polymorphism, Irisin, and Liver Fibrosis in Patients With Nonalcoholic Fatty Liver Disease. *J Clin Endocrinol Metab*.

[66] Polyzos SA, Kountouras J, Anastasilakis AD, Geladari EV, Mantzoros CS (2014). Irisin in patients with nonalcoholic fatty liver disease. *Metabolism*.

[67] Ito M, Suzuki J, Tsujioka S, Sasaki M, Gomori A, Shirakura T (2007). Longitudinal analysis of murine steatohepatitis model induced by chronic exposure to high-fat diet. *Hepatol Res*.

[68] Vonghia L, Ruyssers N, Schrijvers D, Pelckmans P, Michielsen P, De Clerck L (2015). CD4+ROR gamma t++ and Tregs in a Mouse Model of Diet-Induced Nonalcoholic Steatohepatitis. *Mediators Inflamm*.

[69] Toye AA, Lippiat JD, Proks P, Shimomura K, Bentley L, Hugill A (2005). A genetic and physiological study of impaired glucose homeostasis control in C57BL/6J mice. *Diabetologia*.

[70] Ronchi JA, Figueira TR, Ravagnani FG, Oliveira HC, Vercesi AE, Castilho RF (2013). A spontaneous mutation in the nicotinamide nucleotide transhydrogenase gene of C57BL/6J mice results in mitochondrial redox abnormalities. *Free Radic Biol Med*.

[71] Roberts MD, Bayless DS, Company JM, Jenkins NT, Padilla J, Childs TE (2013). Elevated skeletal muscle irisin precursor FNDC5 mRNA in obese OLETF rats. *Metabolism*.

[72] Koide A, Bailey CW, Huang X, Koide S (1998). The fibronectin type III domain as a scaffold for novel binding proteins. *J Mol Biol*.

[73] Michishita E, Park JY, Burneskis JM, Barrett JC, Horikawa I (2005). Evolutionarily conserved and nonconserved cellular localizations and functions of human SIRT proteins. *Mol Biol Cell*.

[74] Lantier L, Williams AS, Hughey CC, Bracy DP, James FD, Ansari MA (2018). SIRT2 knockout exacerbates insulin resistance in high fat-fed mice. *PLoS One*.

[75] Zhang B, Xu D, She L, Wang Z, Yang N, Sun R (2018). Silybin inhibits NLRP3 inflammasome assembly through the NAD(+)/SIRT2 pathway in mice with nonalcoholic fatty liver disease. *FASEB J*.

