# Supplementary material for: NAD+-boosting therapy alleviates nonalcoholic fatty liver disease via stimulating a novel exerkine Fndc5/irisin
Source: Theranostics. 2021 Feb 25;11(9):4381–402. doi: 10.7150/thno.53652 (PMC7977447; doi:10.7150/thno.53652)
Supplement: Supplementary file 1 — Supplementary figures and tables. [file thnov11p4381s1.pdf]

Supplementary Data

Figure S1

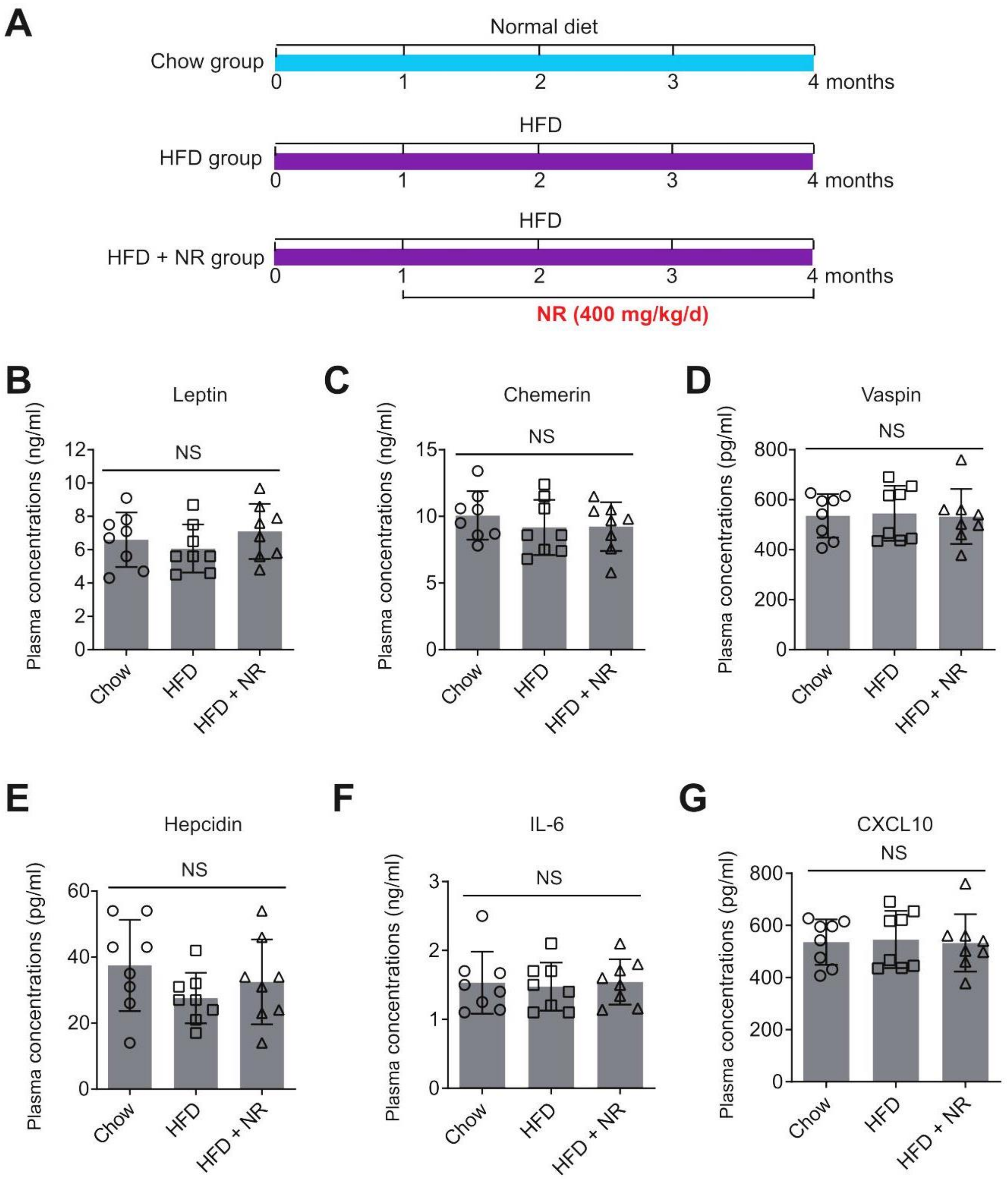

Figure S1. Experimental design of NR therapy in NAFLD mice model and influence of NR on several metabolic-related factors plasma concentrations. (**A**) Experimental design of NR therapy. Mice were fed with HFD for 4 months to induce NAFLD. NR was given at 400 mg/kg/d for 3 months to treat NAFLD. (**B-G**) Plasma concentrations of leptin (**B**), chemerin (**C**), vaspin (**D**), hepcidin (**E**), IL-6 (**F**) and CXCL10 (**G**). NS, no significance.

Figure S2

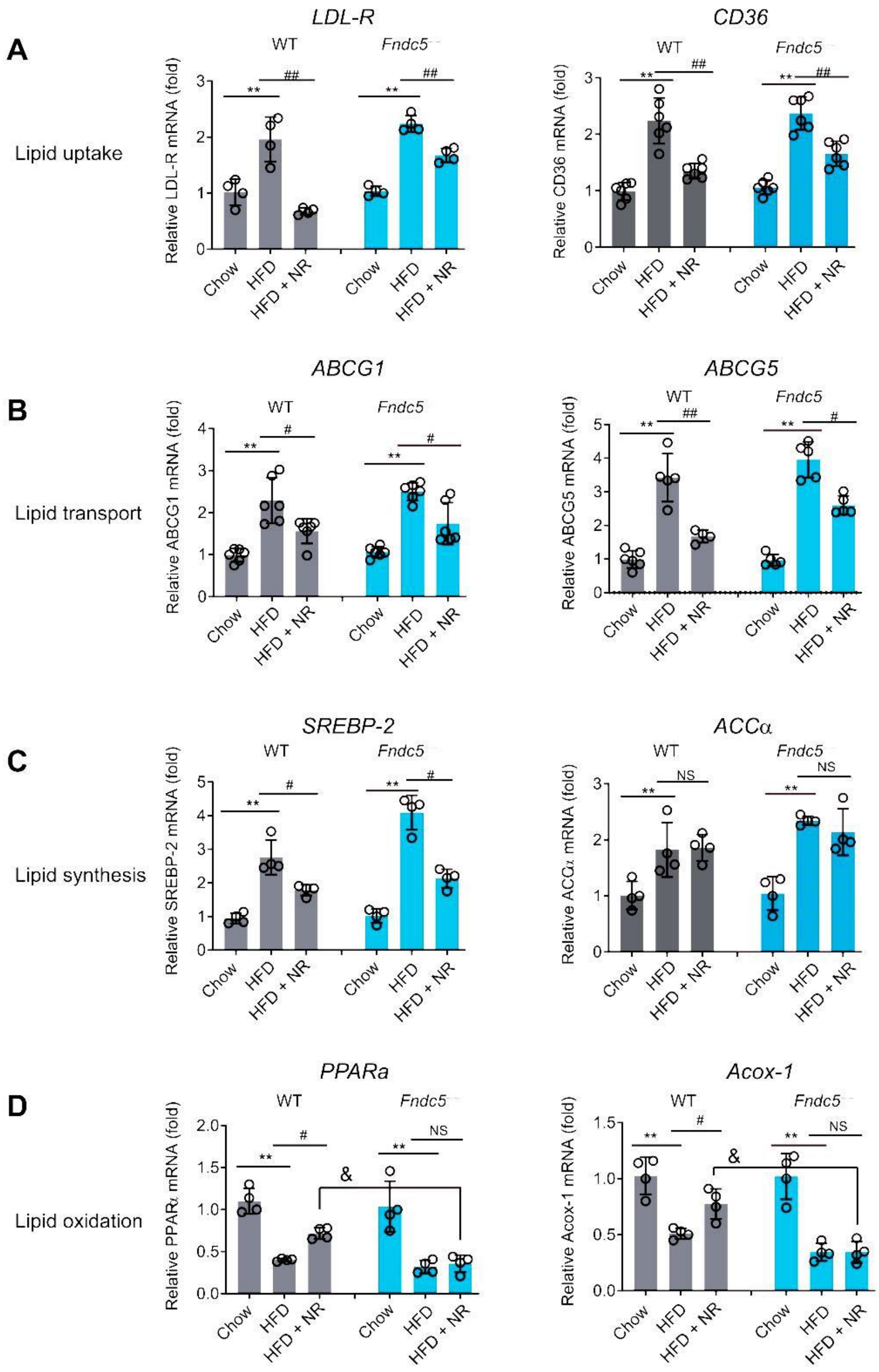

Figure S2. Gene expression of *LDL-R, CD36, ABCG1, ABCG5, SREBP-2, ACCα, PPARa and Acox-1* in liver tissues of WT and *Fndc5*$^{-/-}$ mice fed with HFD and HFD + NR. (A) The expression of lipid uptake genes (*LDL-R* and *CD36*) in liver tissues of WT and *Fndc5*$^{-/-}$ mice. **(B)** The expression of lipid transport genes (*ABCG1* and *ABCG5*) in liver tissues of WT and *Fndc5*$^{-/-}$ mice. **(C)** The expression of lipid synthesis genes (*SREBP-2* and *ACCα*) in liver tissues of WT and *Fndc5*$^{-/-}$ mice. **(D)** The expression of lipid oxidation genes (*PPARα* and *Acox-1*) in liver tissues of WT and *Fndc5*$^{-/-}$ mice. $^{**}P$<0.01 vs chow; $^{\#}P$<0.05, $^{\#\#}P$<0.01 vs HFD; $^{\&}P$<0.01 *Fndc5*$^{-/-}$ vs WT, n = 4-6. NS, no significance.

Figure S3

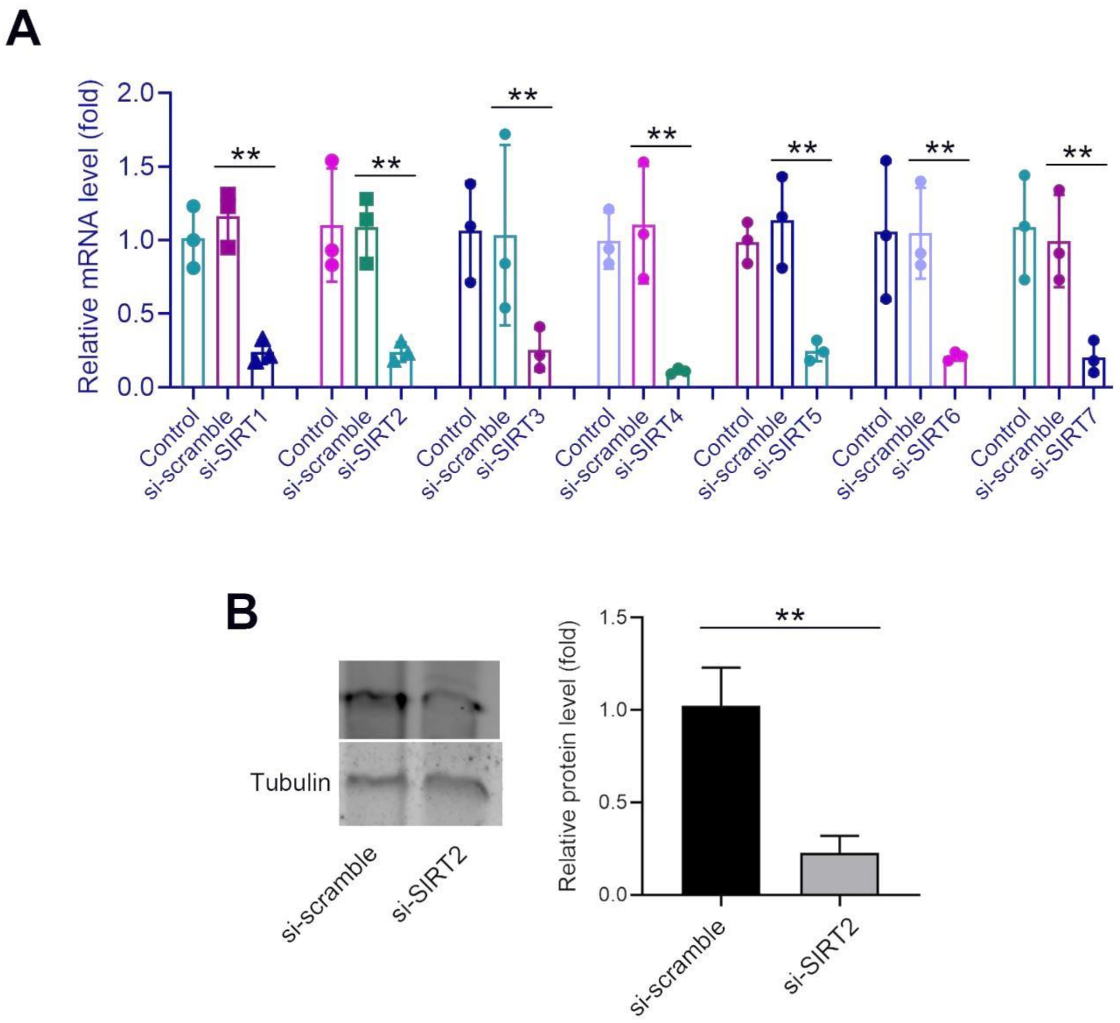

Figure S3. Confirmation of siRNA-mediated knockdown by qPCR and immunoblotting. (A) In the screening experiment, the siRNA-mediated knockdown of SIRT1 to SIRT7 was confirmed by real-time qPCR. **The primers were listed in Supplemental Table 2.** **P<0.01 vs si-scramble. n = 3. **(B)** After discovering SIRT2 might be the linker between NR and Fndc5, the siRNA-mediated knockdown of SIRT2 was further confirmed by immunoblotting. **P<0.01 vs si-scramble. n = 3.

Figure S4

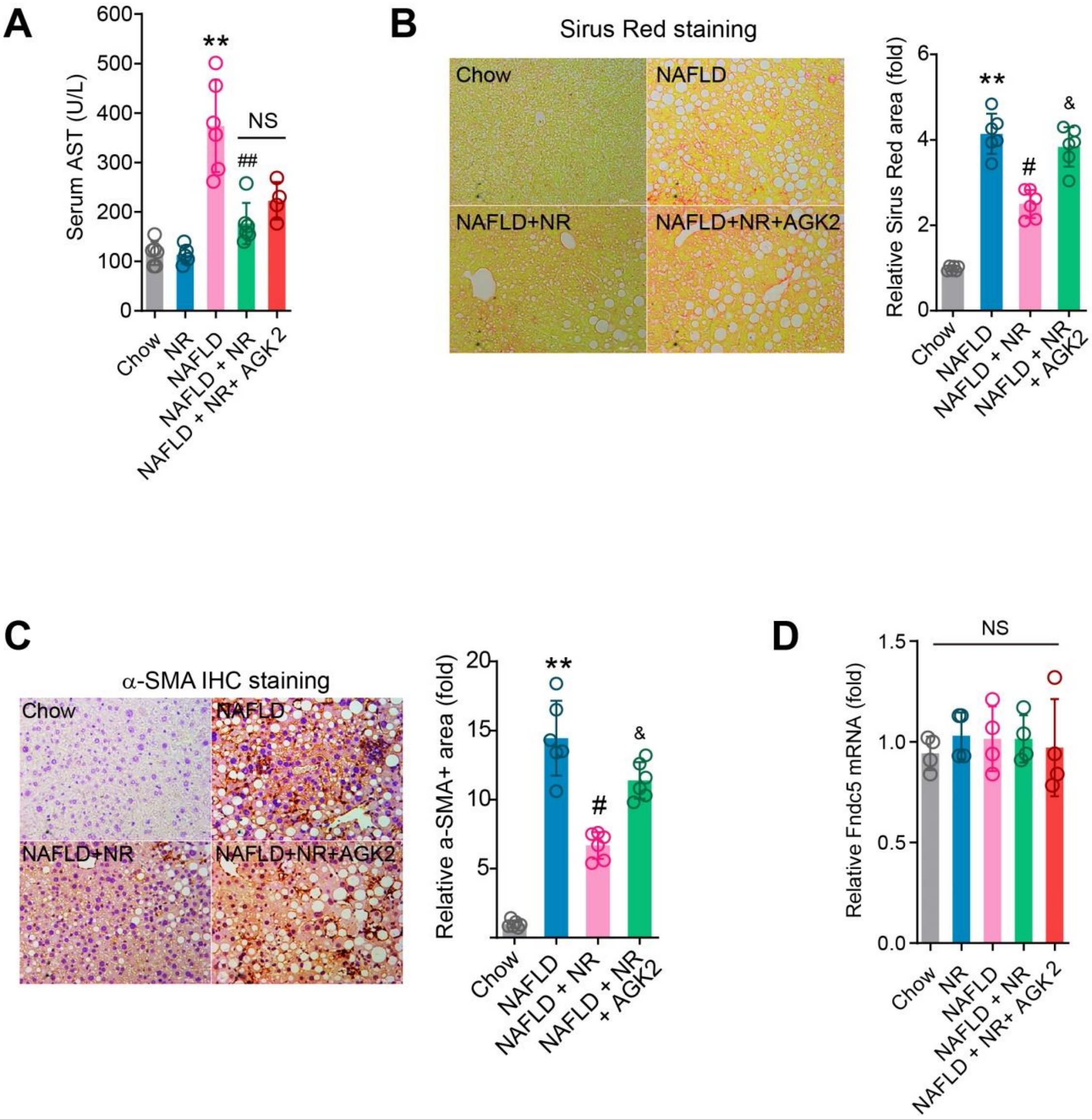

Figure S4. Counteractive effects of SIRT2 inhibitor AGK2 on NR-induced liver protection in MCD-induced NAFLD model. (A) Effects of SIRT2 inhibitor AGK2 on serum AST activity. **P<0.01 vs Chow. $^{\#}P$<0.05 vs NAFLD. n = 6. NS, no significance. **(B)** Sirus Red staining on liver sections in mice. **P<0.01 vs Chow. $^{\#}P$<0.05 vs NAFLD. $^{\&}P$<0.05 vs NAFLD + NR. n = 6. **(C)** Immunohistochemistry staining of α-SMA on liver sections in mice. **P<0.01 vs Chow. $^{\#}P$<0.05 vs NAFLD. $^{\&}P$<0.05 vs NAFLD + NR. **(D)** Quantitative PCR analysis of Fndc5 mRNA level in liver from mice. NS, no significance. Data analyzed by ONE-WAY ANOVA with a Tukey post-test.

Table S1. Antibodies used in this work

Antibody	Source	Catalogue
Anti-Fndc5	Abcam	ab131390
Anti-IRS-1	Santa Cruz Biotechnology	E-12
Anti-p-IRS-1Tyr608	Millipore	09-432
Anti-p-IRS-1Ser307	Cell Signaling Technology	2381
Anti-p-IRS-1Ser636	Novus Biologicals	NB100-82003
Anti-Akt	Cell Signaling Technology	9272
Anti-p-AktThr308	Cell Signaling Technology	13038
Anti-GRP78	Santa Cruz Biotechnology	sc-13968
Anti-CHOP	Santa Cruz Biotechnology	sc-7351
Anti-eIF2α	Santa Cruz Biotechnology	sc-133132
Anti-p-eIF2α	Cell Signaling Technology	3398
Anti-PERK	Santa Cruz Biotechnology	sc-13073
Anti-p-PERK	Cell Signaling Technology	3179
Anti-JNK1/2	Santa Cruz Biotechnology	sc-137019
Anti-p-JNK1/2	Cell Signaling Technology	4668
Anti-ubiquitin	Cell Signaling Technology	3933
Anti-Flag	Sigma-Aldrich	F3165
Anti-Myc	Sigma-Aldrich	SAB1305535
Anti-acetylated-lysine	Cell Signaling Technology	9441
Anti-Caspase-8	Cell Signaling Technology	9746
Anti-HMGB1	Abcam	ab18256
Anti-PGC-1α	Abcam	ab176328
Anti-GAPDH	Proteintech	60004-1-Ig
Anti-Tubulin	Proteintech	66031-1-Ig

Table S2. Sequences of primers for qPCR analysis

Gene	Forward Primer	Reverse Primer
TNF-α	GGAACACGTCGTGGGATAATG	GGCAGACTTTGGATGCTTCTT
IL-6	ATGAAGTTCCTCTCTGCAAGAGAC	CACTAGGTTTGCCGAGTAGATCTC
IL-1β	GAAATGCCACCTTTTGACAGTG	TGGATGCTCTCATCAGGACAG
TFAM	CAAAGGATGATTCGGCTCAG	AAGCTGAATATATGCCTGCTTTTC
PGC-1α	GAAAGGGCCAAACAGAGAGA	GTAAATCACACGGCGCTCTT
NRF1	TGGAGTCCAAGATGCTAATGG	GCGAGGCTGGTTACCACA
Mfn2	CCGATGTGAACCCGTTTCT	AGGCGGAGTCCTCTTCAGC
Mst1	TTGGCAAACTGCATAGCATCC	TCAAATTGGGACTCTCCTTTAGC
NR4A1	GGAAGGCTTAATTGCAGCCA	TTCAGCCTTGTCCATCTGCAT
Bnip3	ATTAATGGCACAGACGCAGC	CCGAACACAGCGTAGATAGACC
ABCA1	GCTGCAGGAATCCAGAGAAT	CATGCACAAGGTCCTGAGAA
ABCG5	AGGGCCTCACATCAACAGAG	GCTGACGCTGTAGGACACAT
CD36	TGTGGGCTCATTGCTGG	TTGATTTTGCTGCTGTTCTTT
LDL-R	AGTGGCCCCGAATCATTGAC	CTAACTAAACACCAGACAGAGGC
SREBP-2	GCAGCAACGGGACCATTCT	CCCCATGACTAAGTCCTTCAACT
ACCa	GAGCCTGAGGAACAGCATCT	CACGAGCCATTCATTATCACTAC
TIMP-1	AGGTGGTCTCGTTGATTCGT	GTAAGGCCTGTAGCTGTGCC
TGFβ-1	TTGCCCTCTACAACCAACACAA	GGCTTGCGACCCACGTAGTA
SIRT1	CGCCTTGCGGTGGACTT	ATGGCTCTATGAAACTGTTCTGGT
SIRT2	CTCAGGATTCAGACTCGGACAC	GCAAGGGCAAAGAAGGGTT
SIRT3	GCCTCTACAGCAACCTTCAGC	CACCCTGTCCGCCATCAC
SIRT4	CGCCCAGCCCTCCTTT	TCCCACCTTTTCTGACCTGTAGT
SIRT5	CCAAGCACATAGCCATCATCTC	CTGCCCTGGTCACGAAGC
SIRT6	ACACCATTCTGGACTGGGAGG	GGCTGTTGGGCTTGGACTTA
SIRT7	TGACGAAGCCTCCAAGCC	CACAACCCCTGCCAAACC
GAPDH	GTATGACTCCACTCACGGCAAA	GGTCTCGCTCCTGGAAGATG

Table S3. Serum parameters of mice fed HFD or HFD+NR after fasting overnight in WT and *Fndc5*$^{-/-}$ mice

Serum parameters	WT (n = 8)			*Fndc5*$^{-/-}$ (n = 8)		
	Chow	HFD	HFD + NR	Chow	HFD	HFD + NR
TC (mg/dL)	154.2±8.2	312.4±11.4**	237.9±3.6**$^{\#\#}$	140.3±7.1	345.5±21.7**	275.1±12.4**$^{\#\#\&}$
LDL-Cho (mg/dL)	74.3±6.1	115.3±8.9**	87.9±3.6**$^{\#\#}$	60.6±5.2	124.5±9.3**	105.1±8.2**$^{\#\&\&}$
HDL-Cho (mg/dL)	66.1±4.5	75.3±5.9**	72.9±7.3	70.5±6.4	84.5±8.4**$^{\&}$	75.1±6.9
TG (mg/dL)	54.6±4.2	115.3±8.9**	87.9±3.6**$^{\#\#}$	60.6±5.2	124.5±9.3**	105.1±8.2**$^{\#\&\&}$
NEFA (mM)	0.16±0.03	0.64±0.12**	0.34±0.06*$^{\#\#}$	0.14±0.02	0.88±0.09**$^{\&}$	0.67±0.07**$^{\#\&\&}$

TC, total cholesterol; TG, triglyceride; NEFA, non-esterified fatty acid. *P<0.05, **P<0.01 vs Chow; $^{\#}P$<0.05, $^{\#\#}P$<0.01 vs HFD; $^{\&}P$<0.05, $^{\&\&}P$<0.01 vs *Fndc5*$^{-/-}$ vs WT.

Supplemental Methods

Hepatic cholesterol and triglycerides determination

Liver tissues were homogenized in ice-cold 20 mM Tris buffer (pH 7.4). The cholesterol and triglycerides in liver tissues were measured using commercial kits (No. 10007640 and No. 10010303, Cayman Chemical, Ann Arbor, MI) respectively.

Immunohistochemistry and apoptosis assay

For immunohistochemistry experiments, frozen 8-μm-thick sections were fixed in 4% paraformaldehyde and dried in 37oC for 24 hours. Then, the sections were blocked by 5% BSA solution for 1 hour and incubated in primary antibodies dissolved in 1% Tris buffered saline with Tween 20 (TBST) overnight at 4 oC. The sections were then washed in TBST three times and incubated with horseradish peroxidase-conjugated secondary antibodies. Staining is visualized using substrate diaminobenzidine. Images were obtained in Olympus BX51 microscope (Tokyo, Japan, Leica). The following antibodies were used: F4/80 (LS-C96373-100, Lifespan, 1:500 dilution), CD11b (ab6672, Abcam, 1: 1000 dilution) and -SMA (ab7817, Abcam, 1: 500 dilution). Immunofluorescence TUNEL assay was used to assess apoptosis. Tissue sections were placed and fixed in 4% paraformaldehyde, and incubated with immunofluorescent TUNEL reaction mixture for 1 h in box. DAPI was used to stain nuclei. Immunofluorescence images were obtained in Olympus IX71 microscope (Tokyo, Japan, Leica).

Immunoblotting

Tissues were homogenized with the RIPA buffer (Beyotime, Haimen, China) supplemented with a protease/phosphatase inhibitor cocktail (Pierce Technology, Rockford, IL). The samples were boiled, separated by SDS–PAGE, transferred to a polyvinylidene difluoride membrane (Millipore) and blotted with specific primary antibodies. The membranes were washed by phosphate buffered saline with Tween 20 for three times and then incubated with Infrared-Dyes-conjugated secondary antibodies (Li-Cor, Lincoln, NE). The images were obtained with Odyssey Infrared Fluorescence Imaging System (Li-Cor). All immunoblotting

experiments were repeated at least three times.

Immunoprecipitation

Cells were lysed in radioimmunoprecipitation assay buffer (50 mM Tris-HCl [pH 7.4], 50 mM NaCl, 1% NP-40, 1% sodium deoxycholate, and 0.1%–1% SDS) supplemented with protease inhibitor cocktail tablets (G6521, Promega) to minimize protein degradation. Stringent RIPA buffer (1% SDS) was used in IP experiments to ensure clean protein immunoprecipitation, while mild RIPA buffer (0.1% SDS) was used in co-IP experiments for protein-protein interaction assays. Cell lysates were centrifuged at 13,000 rpm for 20 min. The supernatant was subjected to immunoprecipitation with protein A/G-agarose beads (Santa Cruz Biotechnology) and followed indicated antibodies. For a clean immunoprecipitation, the beads rinsed in NP-40 buffer with vortexing (25 mM Tris-HCl [pH 7.4], 150 mM NaCl, 1 mM EDTA, and 1% NP-40) for three times. Then, the agarose beads were boiled with loading buffer and proteins were separated by electrophoresis on 12% SDS–PAGE and followed by immunoblotting analysis with specific antibodies or control IgG.

Mitochondrial Complex I, II and IV activity and NAD^+/NADH ratio

Liver tissues were homogenized with distilled water. Mitochondrion was isolated using a commercial kit from Beyotime Institute of Biotechnology (Haimen, China). The mitochondrial complex I, II and IV activity were analyzed using commercial kits (№ 700930, 700940 and 700990 respectively) from Cayman Chemical (Ann Arbor, MI) according the instructions from the manufacturer. NAD^+/NADH ratio was determined with EZScreen™ NAD^+/NADH Colorimetric Assay Kit (#K958, BioVision).

Blood and plasma parameters

At the endpoint of the experiment, mice were anesthetized with an intraperitoneal injection of sodium pentobarbital (50 mg/kg). Blood was obtained from the right atrium and allowed to clot in an upright position for at least 30 minutes and then centrifuged at 500 rpm for 10 minutes to obtain plasma. Blood glucose values were determined using an automatic glucose

monitor (One Touch® Ultra, Johnson & Johnson, Milpitas, CA). Plasma insulin level was determined using a commercial sandwich ELISA kit (LINCO research, MI) as described. The plasma ALT, AST and ALP levels were used to gauge hepatic dysfunction and determined with an automatic biochemistry analyzer (Beckman Coulter, Miami, FL) according to the manufacturer's instructions.
